# Comparative effectiveness of telemedicine strategies on type 2 diabetes management: A systematic review and network meta-analysis

**DOI:** 10.1038/s41598-017-12987-z

**Published:** 2017-10-04

**Authors:** Shaun Wen Huey Lee, Carina Ka Yee Chan, Siew Siang Chua, Nathorn Chaiyakunapruk

**Affiliations:** 1 School of Pharmacy, Monash University Malaysia, Bandar Sunway, Malaysia; 2 Asian Centre for Evidence Synthesis in Population, Implementation and Clinical Outcomes (PICO), Health and Well-being Cluster, Global Asia in the 21st Century (GA21) Platform, Monash University Malaysia, Bandar Sunway, Selangor Malaysia; 3 School of Psychology, Australian Catholic University, Brisbane, Australia; 4 Department of Pharmacy, Faculty of Medicine, University of Malaya, Kuala Lumpur, Malaysia; 5 School of Pharmacy, Faculty of Health and Medical Sciences, Taylor’s University, Lakeside Campus, Subang Jaya, Malaysia; 6 Center of Pharmaceutical Outcomes Research (CPOR), Department of Pharmacy Practice, Faculty of Pharmaceutical Sciences, Naresuan University, Phitsanulok, Thailand

## Abstract

The effects of telemedicine strategies on the management of diabetes is not clear. This study aimed to investigate the impact of different telemedicine strategies on glycaemic control management of type 2 diabetes patients. A search was performed in 6 databases from inception until September 2016 for randomized controlled studies that examined the use of telemedicine in adults with type 2 diabetes. Studies were independently extracted and classified according to the following telemedicine strategies: teleeducation, telemonitoring, telecase-management, telementoring and teleconsultation. Traditional and network meta-analysis were performed to estimate the relative treatment effects. A total of 107 studies involving 20,501 participants were included. Over a median of 6 months follow-up, telemedicine reduced haemoglobin A1c (HbA1c) by a mean of 0.43% (95% CI: −0.64% to −0.21%). Network meta-analysis showed that all telemedicine strategies were effective in reducing HbA1c significantly compared to usual care except for telecase-management and telementoring, with mean difference ranging from 0.37% and 0.71%. Ranking indicated that teleconsultation was the most effective telemedicine strategy, followed by telecase-management plus telemonitoring, and finally teleeducation plus telecase-management. The review indicates that most telemedicine strategies can be useful, either as an adjunct or to replace usual care, leading to clinically meaningful reduction in HbA1c.

## Introduction

Diabetes mellitus is fast becoming a global public health challenge. It is estimated that more than 415 million people worldwide have diabetes, representing nearly 9.1% of the global adult population, and this number is expected to increase by more than half to 642 million by the year 2040^1^. Diabetes is no longer a disease of the affluent, as nearly 77% of the world’s diabetes population lives in low and middle-income countries, and consumes nearly USD 548 billion in health care expenditure globally^2,3^.

To combat this pandemic, a multi-faceted approach is necessary. Currently, various policy initiatives have been suggested to cope with this transformation. These include a comprehensive framework for diabetes prevention, integrating individual-level factors with environmental and policy factors at a macro-level^4^. At the patient level, a coordinated approach which integrates the beneficial effects of monitoring, education and support by health care professionals is needed. Telemedical care, or the use of telecommunication for healthcare delivery holds the promise to complement medical management in people with diabetes since it could facilitate early detection, diagnosis, monitor disease progression and management^5–8^. These can range from simple remote monitoring systems^9^ to more complex Web-based systems which can coordinate, manage and educate patients^10^.

Previous reviews of telemedicine studies in diabetes have been published, but most of them focused on specific telemedicine interventions such as telemonitoring^11^, teleconsultation^6^ or using interventions delivered by computer^12^. This has delayed potential translation of evidence to changes in policy and practice. This study aimed to review the literature regarding telemedicine comprehensively and determine the relative effectiveness of the various telemedicine technologies in an outpatient setting.

## Methods

### Literature search

A systematic review was conducted on the following databases since inception until September 30, 2016: MEDLINE, EMBASE, PsycINFO, Cochrane Central Register of Controlled Trials, Web of Science, and CINAHL Plus, using a combination of keywords and MeSH terms. This was supplemented with manual search for citations in reference lists of retrieved articles, textbooks, grey literature as well as conference abstracts (Supplemental Table S1). The full list of databases and search terms used are presented in the Appendix and documented online (PROSPERO registry CRD42015023913).

### Eligibility criteria

Studies were included if they were: (1) randomized controlled studies (RCT); (2) examined the use of telemedicine, defined as the use of medical exchange between different sites via electronic communications to improve patients’ health status; (3) conducted in ambulatory type 2 diabetes patients; and (4) generated content to improve one or more diabetes self-management domains through feedback, advice, reinforcement, goal setting or patient decision support.

### Data extraction

Data were independently extracted by two reviewers and any discrepancies were resolved through adjudication. Information extracted included study characteristics, study participants, eligibility criteria, as well as details of intervention and outcome measures. When more than one comparison group was evaluated in the RCT, data for the most intensive treatment were extracted. Most of the reported outcomes were reported as mean changes, but in some cases as pre-post intervention or percentage of change. In the latter cases, the pre-post intervention standard deviations along with a correlation were used to derive the difference along with their standard deviations. For assessment of study quality, the Cochrane risk of bias tool^13^ was used.

### Content of intervention and control conditions

Since a wide variety of interventions had been tested with the goal of improving quality of care among type 2 diabetes patients, the interventions were characterized according to their telemedicine strategies as described in Table 1, based upon an adaptations from the American Telemedicine Association^14^, Tricco *et al*.^15^ and Lee *et al*.^16^.

**Table 1:** Taxonomy of classification used in the current study.

Telemedicine strategy targeting patient
**Teleeducation**
Any intervention that utilizes application of information and communication technologies (e.g. telephone lines, internet) for the delivery of distance learning, teaching or training to remote participants.
**Teleconsultation**
Two way communication between a health care provider / specialist and patients or between clinicians using a range of communication and information technologies (email, phone, automated messaging system, Internet or other equipment without face-to-face contact) that aim to provide health care at a distance. The interaction is directed at patient care from the clinician and communication is interactive and occurs within an episode of care.
**Telemonitoring**
Any process which uses an audio, video or telecommunication and electronic information to monitor health status of a patient from a distance which is then transmitted back to the clinician. This strategy is strictly based on clinical data and excludes clinical skills.
**Telecase-management**
A collaborative approach that focuses on the coordination, integration and direct delivery of beneficiary services provided in collaboration with or supplementary to primary care for improving the efficiency, depth or breadth of clinical care.
**Telementoring**
Process of using either audio, video or any telecommunication and electronic information processing technology by a person who has gone through a specific experience to provide individual guidance, mentorship or direction to another person who is new to the experience.

In addition, interventions were also coded based on the technology used to support the care delivery process and psychological intervention based on the taxonomy described by Welton^17^ (Supplemental Table S2).

### Outcomes and effect modifiers

The primary outcome of interest was glycaemic control based on the absolute change in HbA1c from baseline to end of study. Secondary outcomes included changes in cardiovascular risk factors (blood pressure, serum lipid profiles, fasting blood glucose and body weight). The study characteristics were also considered as potential effect modifiers in the current study, including sample size, study location, delivery mechanisms, study duration as well as baseline HbA1c.

### Statistical analyses

For studies that provided sufficient information on glycaemic control (HbA1c or fasting plasma glucose), a permutation based meta-analysis with random effects model^18^ was performed. This permutation test was chosen since the method has been found to be useful to reduce the false-positive rates as significant heterogeneity is expected as well as the large number of covariates which are expected to be effect modifiers in this study^19^. Statistical heterogeneity was evaluated by using the *I* ^2^ statistics^20^. When heterogeneity was present, a random-effects meta-regression analysis^21^ was performed to take into account the clinical (e.g. baseline HbA1c, duration of study, study year) and methodological variations (e.g. type of telemedicine, mode of telemedicine delivery, medication changes allowed) which could affect the effects of telemedicine on HbA1c.

Subsequently, a multivariable model was constructed, and the goodness-of-fit was determined for each model using the adjusted R^2^, denoting the proportion of between-study-variation explained by the covariates^21^. The variables investigated in the model as possible sources of inconsistencies and/or heterogeneity are: age of publication, number of participants, location of study (by region), study duration as well as baseline mean HbA1c of all participants at entry. In the analysis, the dependent (outcome variable) was the study-level effect size or mean difference of HbA1c between the intervention and the control arms. For all other variables that were imputed into the model, the mean of each variables were used in the meta-regression analyses.

A network meta-analysis was conducted which combined direct and indirect effects of various treatments using the *network* command in STATA^22–25^. This allowed the simultaneous comparison of several interventions, thus increasing precision and also to evaluate their relative effectiveness, using the resampling-based surface under the cumulative ranking curve (SUCRA) and mean ranks^26^. The results were subsequently presented with 95% confidence intervals (CIs)^26^. Inconsistency checks were performed for closed loop in the network^27^. This was performed based upon the assumption of a common heterogeneity parameter across all loops in the network as derived from the network meta-analysis model. We also ran the design-by-treatment interaction model to assess the presence of inconsistencies across the entire network^23^. Publication bias was evaluated visually using funnel plots and quantified. Forest plots were used to summarize pooled treatment comparison and comparison-adjusted funnel plots for small study effects. All analyses were performed using Stata software version 13.0 (Stata Inc, College Station, Texas).

## Results

### Description of studies

Information from the database search yielded 6,660 potentially relevant studies, of which 209 full-text were assessed for eligibility. One hundred and seven publications, which included 20,501 randomly assigned participants fulfilled the inclusion criteria and were included in the current review (Supplemental Figure S1). All studies were published between 1998 and 2016, and a large proportion had fewer than 100 participants (*n* = 38, 36%). The reported mean age of participants was between 42 to 71 years, mean duration of type 2 diabetes ranged from 2 to 24 years and median HbA1c was 8.1% at baseline. More than half of these studies were conducted in North America (50.5%) and most studies included both male and female participants. Majority of the studies were only for short term, with a median follow-up period of 6 months or less (66.7%).

These telemedicine interventions varied as the trials had used a variety of platform for communication and delivery of intervention, including telephone (42%), the internet (34%), mobile phones (13%), SMS (9%), video conferencing (7%), computer (4%) as well as pagers (1%). These studies had incorporated elements of behavioural therapy (90%), educational counselling (84%), psychosocial support (21%; e.g. peers), cognitive therapy (17%) or financial incentives (3%) as part of the intervention component (Table 2, Supplemental Tables S3–S7). Intervention providers include nurses (48%), physicians (17%), allied healthcare professionals (15%; pharmacist, dieticians), as well as non-specialised support workers (19%; including lay person, social workers and community health care workers). In addition, the definition of usual care provided by each of these studies also varied. Many of these studies, usual care was defined as regular follow-up or practice of the particular site. In most of these studies, they include general diabetes education (29%) and self-monitoring of blood glucose (9%). The details of usual care provided to participants was not reported in 52% of trials. Full details of the components for usual care/control and intervention of each study can be found in Supplemental Tables S3–S7.

**Table 2:** Baseline characteristics of included studies. Values are number of studies unless otherwise stated.

Eligible studies	
No. of studies	107
Median (IQR) study duration (months)	6 (6–12)
**Participant demographics**	
Total participants	20,467
Median (IQR) age (years)	57 (53–61)
Median (IQR) duration of diabetes (years)	9·6 (7·8–12·3)
No. of male participants, n (%)	8,564 (50·4)
Median (IQR) HbA1c (%)	8·1 (7·6–9·0)
**Study location, n**	
North America	54
Europe	16
Asia	34
Australia	2
South America	1
**Intervention setting†**	
Community-based	31
Primary care based	56
Specialist setting / Hospital setting based	36
**Telemedicine strategies**	
Teleconsultation	8
Tele-education	29
Telecase-management	10
Telemonitoring	16
Telementoring	11
Tele-education & Telecase-management	12
Tele-education & Telemonitoring	8
Tele-management & Telemonitoring	12
Tele-management & Teleconsultation	1
Study outcome examined*	
HbA1c	94 (88%)
Fasting plasma glucose	24 (22%)
Total cholesterol	32 (30%)
Low density lipoprotein	32 (30%)
High density lipoprotein	31 (29%)
Triglycerides	31 (29%)
Systolic blood pressure	36 (34%)
Diastolic blood pressure	30 (28%)
Body mass index	27 (25%)
Medium of communication used*	
Short message service	9
Telephone	44
Internet	38
Mobile phone	14
Video conferencing system	7
Computer	4
Pager	1
Administrator of intervention*	
Primary care physician	20
Nurse	50
Pharmacist	5
Psychologist	1
Endocrinologist	6
Diabetes educator	16
Dietician / Nutritionist	11
Others – lay person, social worker	20
Psychological model used in intervention*	
Behavioural therapy	97
Educational intervention	89
Cognitive therapy	18
Psychosocial support	22
Financial incentive	3

IQR- Interquartile range, *Some studies may report the same strategy multiple times. ^†^Some studies reported conducting the study in different intervention sites.

In terms of risk of bias assessment, 67 (64%) studies had adequate reporting on sequence generation, 69 (66%) studies described loss to follow-up, while only 40 (38%) studies reported intention-to-treat population in their analyses (Supplemental Figure S2).

### Primary outcome

#### Pairwise meta-analysis

Telemedicine was superior to usual care in improving HbA1c levels, with a mean difference (MD) of −0.43% (95% CI: −0.64% to −0.21%; *p* < 0.001), but there was substantial heterogeneity (*Q* = 88,052, *I* ^2^ = 99.9%, H^2^ = 966; *p* < 0.001). Subgroup analysis revealed that larger effects were observed in studies with shorter intervention duration (≤3 months: −0.65% [−0.91% to −0.39%]; 4–6 months: −0.38% [−0.85 to 0.09%]; 7–12 months: −0.62% [−0.91% to −0.34%]; and −0.23% [−0.35% to −0.11%], Table 3).

**Table 3:** Results of pairwise meta-analysis for primary and secondary outcomes of different telemedicine strategies.

Comparison		No. of studies	No. of patients	*I* ^2^ [H^2^]*	Pairwise meta-analysis mean difference (95% CI)
**Glycosylated haemoglobin (%)**					
Usual care vs	Telemonitoring	14	1,577	71% [2]	−0.44 (−1.63 to −0.07)
	Teleconsultation	7	1,328	98% [44]	−0.64 (−3.74 to −0.02)
	Tele-education	26	4,211	81% [4]	−0.36 (−0.97 to −0.07)
	Telecase-management	8	2,620	97% [28]	−0.28 (−2.87 to 0.13)
	Telementoring	11	2,892	99% [253]	−0.28 (−1.51 to 0.08)
	Tele-education & telemonitoring	8	1,540	72% [2]	−0.35 (−2.20 to −0.02)
	Telecase-management & telemonitoring	9	1,194	84% [5]	−0.54 (−2.44 to −0.06)
	Tele-education & telecase-management	9	1,409	96% [26]	−0.31 (−2.66 to −0.02)
	Telecase-management & Teleconsultation	1	40	NA [NA]	−1.20 (−2.30 to −0.10)
Teleeducation vs	Telecase-management	1	100	NA [NA]	−0.40 (−1.03 to 0.23)
	Tele-education & telecase-management	1	79	NA [NA]	−0.16 (−0.70 to 0.38)
Telemonitoring vs	Tele-education & telemonitoring	1	152	NA [NA]	0.02 (−0.37 to 0.41)
**Fasting plasma glucose (mmol/L)**					
Usual care vs	Teleeducation	7	1393	79% [4]	−0.67 (−1.23 to −0.11)
	Telecase-management	3	494	61% [NA]	−1.78 (−2.84 to −0.72)
	Telemonitoring	7	566	41% [1]	−0.90 (−1.32 to −0.49)
	Telecase-management & Telemonitoring	3	167	0% [NA]	−1.69 (−2.46 to −0.93)
	Teleeducation & Telecase-management	3	155	0% [NA]	0.04 (−0.79 to 0.87)
	Teleeducation & Telemonitoring	1	100	NA [NA]	−0.70 (−1.60 to 0.20)
**Total cholesterol (mmol/L)**					
Usual care vs	Teleeducation	11	2,517	40% [1]	−0.12 (−0.24 to 0.00)
	Telecase-management	3	1,628	82% [NA]	−0.02 (−0.47 to 0.43)
	Telemonitoring	9	965	94% [15]	0.00 (−0.92 to 0.28)
	Telementoring	3	859	0% [NA]	0.03 (0.02 to 0.04)
	Teleeducation & Telemonitoring	1	100	NA [NA]	−0.27 (−0.57 to 0.03)
	Telecase-management & Telemonitoring	2	339	0% [NA]	−0.22 (−0.42 to −0.01)
	Teleeducation & Telecase-management	2	117	84% [NA]	0.14 (−0.78 to 1.06)
Teleeducation vs	Telecase-management	1	100	NA [NA]	−0.08 (−0.45 to 0.29)
**High-density lipoprotein (mmol/L)**					
Usual care vs	Teleeducation	10	1,593	35% [1]	0.01 (−0.02 to 0.03)
	Telecase-management	2	246	0% [NA]	0.01 (−0.02 to 0.05)
	Telemonitoring	9	938	15% [1]	0.00 (−0.11 to 0.03)
	Teleconsultation	2	84	95% [NA]	0.48 (−0.39 to 1.35)
	Telementoring	2	762	99% [NA]	−0.06 (−0.20 to 0.08)
	Teleeducation & Telecase-management	1	53	NA [NA]	0.10 (−0.07 to 0.27)
	Telecase-management & Telemonitoring	4	472	57% [NA]	−0.13 (−0.26 to −0.01)
Teleeducation vs	Telecase-management	1	100	NA [NA]	−0.03 (−0.15 to 0.09)
**Low-density lipoprotein (mmol/L)**					
	Teleeducation	8	1,768	46% [1]	0.00 (−0.05 to 3.67)
	Telecase-management	4	1,923	91% [NA]	0.08 (−0.22 to 0.37)
	Teleconsultation	3	1,145	96% [NA]	−0.19 (−0.47 to 0.09)
	Telemonitoring	8	861	71% [2]	−0.07 (−0.17 to 0.19)
	Telementoring	3	1,091	99% [NA]	−0.06 (−0.22 to 0.11)
	Telecase-management & Telemonitoring	4	472	15% [NA]	−0.06 (−0.24 to 0.12)
	Teleeducation & Telecase-management	1	359	NA [NA]	0.03 (0.02 to 0.04)
Teleeducation vs	Telecase-management	1	100	NA [NA]	−0.09 (−0.39 to 0.21)
**Triglycerides (mmol/L)**					
	Teleeducation	9	1,928	39% [1]	−0.14 (−0.30 to −0.06)
	Telecase-management	2	246	0% [NA]	0.04 (−0.07 to 0.15)
	Teleconsultation	2	84	NA [NA]	−0.08 (−0.36 to 0.19)
	Telemonitoring	9	938	39% [1]	−0.04 (−0.14 to 0.63)
	Telementoring	1	628	NA [NA]	0.20 (0.15 to 0.25)
	Teleeducation & Telemonitoring	1	100	NA [NA]	−0.17 (−0.54 to 0.20)
	Telecase-management & Telemonitoring	3	293	0% [NA]	−0.24 (−0.50 to 0.02)
	Teleeducation & Telecase-management	2	117	0% [NA]	−0.07 (−0.54 to 0.39)
Teleeducation vs	Telecase-management	1	100	NA [NA]	−0.02 (−0.50 to 0.46)
**BMI (kg/m** ^**2**^ **)**					
	Teleeducation	6	1,256	0% [0]	0.00 (−0.08 to 0.07)
	Telecase-management	3	687	40% [NA]	1.10 (0.22 to 1.98)
	Teleconsultation	3	611	40% [NA]	0.26 (−0.65 to 1.18)
	Telemonitoring	6	778	91% [11]	−0.98 (−2.26 to 0.31)
	Telementoring	4	1,188	99% [NA]	−0.40 (−1.32 to 0.52)
	Teleeducation & Telemonitoring	3	261	50% [NA]	−0.60 (−1.74 to 0.54)
	Telecase-management & Telemonitoring	3	335	0% [NA]	0.14 (−0.23 to 0.52)
	Teleeducation & Telecase-management	2	247	0% [NA]	0.08 (−1.24 to 1.40)
Teleeducation vs	Telecase-management	1	100	NA [NA]	0.00 (−2.53 to 2.53)
**Systolic blood pressure (mm Hg)**					
	Teleeducation	7	1,791	0% [0]	−4.05 (−5.64 to −1.10)
	Telecase-management	3	1,785	0% [NA]	−2.65 (−4.62 to −0.68)
	Teleconsultation	3	1,145	88% [NA]	−0.29 (−2.68 to 2.10)
	Telemonitoring	6	883	0% [0]	−0.03 (−1.03 to 0.00)
	Telementoring	5	1,536	96% [NA]	0.89 (−0.64 to 2.43)
	Teleeducation & Telemonitoring	4	709	78% [NA]	−3.91 (−10.07 to 2.25)
	Telecase-management & Telemonitoring	5	520	0% [NA]	−2.16 (−5.22 to 0.91)
	Teleeducation & Telecase-management	2	574	88% [NA]	−0.74 (−8.81 to 7.33)
Teleeducation vs	Telecase-management	1	100	NA [NA]	−5.76 (−12.15 to 0.63)
**Diastolic blood pressure (mm Hg)**					
	Teleeducation	6	1,751	56% [1]	−1.93 (−4.04 to −0.22)
	Telecase-management	3	1,785	0% [NA]	−1.51 (−2.45 to −0.57)
	Teleconsultation	2	510	88% [NA]	−3.72 (−8.80 to 1.36)
	Telemonitoring	5	658	40% [NA]	−0.97 (−2.91 to 0.97)
	Telementoring	4	1,237	98% [NA]	1.16 (−0.13 to 2.45)
	Teleeducation & Telemonitoring	3	509	17% [NA]	−0.82 (−2.94 to 1.30)
	Telecase-management & Telemonitoring	5	520	0% [NA]	−1.72 (−3.59 to 0.15)
	Teleeducation & Telecase-management	1	215	NA [NA]	3.00 (−0.10 to 6.10)
Teleeducation vs	Telecase-management	1	100	NA [NA]	−3.30 (−8.33 to 1.73)
**Quality of life (Problem Areas in Diabetes)**					
	Intervention	2	194	53% [NA]	−2.18 (−10.28 to 5.92)
**Hypogylcaemia (% of patients affected)**					
	Intervention	2	101	80%	−0.20 (−0.57 to 0.17)*
**Diabetes self-efficacy Scale (DSES)**					
	Intervention	3	945	58% [NA]	0.46 (−0.05 to 0.97)

*Mean difference.

Overall, heterogeneity in the pairwise meta-analysis was high and thus meta-regression analyses were conducted to determine the sources of heterogeneity (see below). No statistically significant inconsistency was indicated in most loops within the network for all outcomes examined.

### Moderation of effectiveness of study features and intervention mechanisms

In studies which were conducted in Asia, a greater reduction in HbA1c values was noted compared to studies conducted in Europe or North America (MD: −0.84% vs. −0.20%, *p* < 0.001). Effect sizes were not significantly different when other key features (e.g. baseline HbA1c, study sample size) or interventional content (e.g. interactive vs. non-interactive, different psychological intervention content) were examined (Table 3, Supplemental Table S8). Similarly, none of the items from the Cochrane risk of bias tool were significant effect modifier but studies that reported high loss to follow-up (≥20%) had smaller MD in HbA1c Ncompared to trials with low loss to follow-up (<20%).

Since the providers of intervention (e.g. doctor, nurse) and study duration were reported in less than 5 trials of different telemedicine platform, it was not possible to use the meta-regression to evaluate the merits of these covariates.

### Network meta-analyses

**Figure 1:**
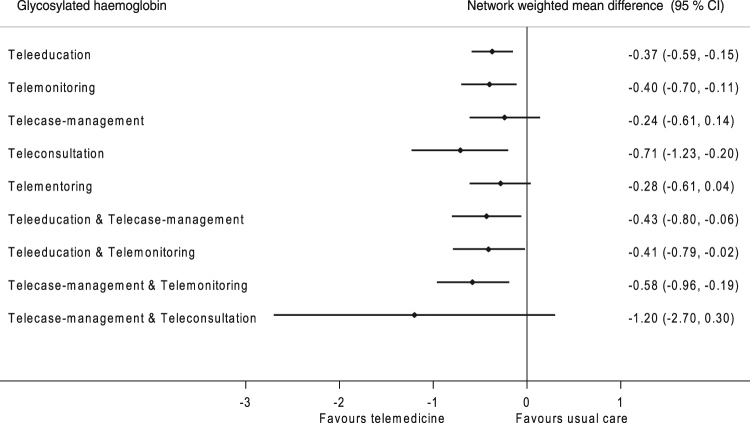
Network meta-analysis estimates of change in glycated haemoglobin in adults with type 2 diabetes.

Ninety-three trials (16,978 participants) contributed to the network meta-analyses (Figure S3a, appendix). All telemedicine strategies except telementoring and telecase-management reduced HbA1c significantly compared to usual care (Fig. 1). However, none of the strategies were significantly better than another (Supplemental Table S9). When the strategies were ranked according to their effectiveness using the surface under cumulative ranking curve statistics, the three most effective interventions were: teleconsultation alone, followed by telecase-management plus telemonitoring and teleeducation plus telecase-management. Comparison-adjusted funnel plots for the network meta-analysis indicated very little evidence of asymmetry (Supplemental Figures S4–S5).

### Secondary outcomes

**Figure 2:**
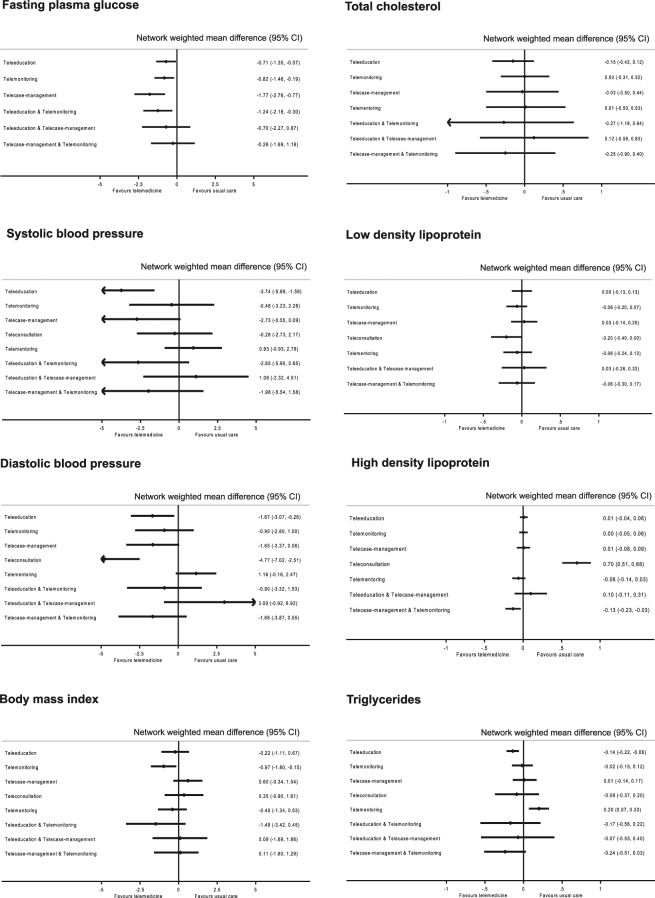
Network meta-analysis estimates of changes in secondary cardiovascular outcomes of different telemedicine strategies compared to usual care.

Figure 2 presents the estimated effects of different telemedicine strategies on cardiovascular outcomes. Across all outcomes examined, point estimates indicated that most telemedicine strategies were generally similar to usual care (Supplemental Tables S8–S9). Five studies reported a decrease in the frequency of hypoglycaemia^7,28–31^ in the telemedicine group, while one study reported an increase^32^. There was no evidence that telemedicine reduced the risk of hypoglycaemia (risk differences: −0.20%; 95 CI: −0.57% to 0.17%). In all nine studies which reported quality of life, no significant change (using either generic quality of life scale or diabetes specific quality of life scale) between groups were reported^3,33–40^. Similarly, pooled analysis showed no improvement in the quality of life in the Problem Area in Diabetes scale (MD: −2.18; −10.28 to 5.92).

### Publication bias and Sensitivity analyses

Omission of each study sequentially did not lead to a significant change in the estimates of both primary as well as secondary outcomes, except for the exclusion of study by Shahid *et al*.^41^, which reduced the effectiveness of telemedicine in reducing HbA1c from −0.43% to −0.34% (95%CI: −0.41 to −0.27). Repeated analysis with fixed effect model did not significantly alter the results or heterogeneity (Supplemental Figure S5 and Table S10). No significant bias was observed using the Egger regression analysis (p = 0.36) but Begg rank-correlation showed evidence of small study effects (p < 0.01).

## Discussion

This study is the largest and most comprehensive structured review to date on the effects of telemedicine. It presents evidence on the relative effectiveness of various telemedicine strategies for individuals with type 2 diabetes as well as factors which are associated with improved patient outcomes and those that do not. Despite the presence of substantial heterogeneity, telemedicine interventions were found to produce significantly better glycaemic outcomes than usual care using pairwise meta-analyses, and some of the changes were of clinical significance. This was similarly confirmed in the network meta-analysis. However, we did not note any significant effects on other outcomes such as quality of life, risk of hypoglycaemia, blood pressure and blood cholesterol.

The biggest potential of telemedicine lies in its ability to help people who cannot be accommodated with existing healthcare clinics due to geographical constraints or resources^42^. Results of our study suggest that telemedicine produced clinically meaningful reduction in HbA1c^43^, with an average reduction of between 0.37% and 0.71%, depending on which strategies are used. Studies have shown that a 1% drop in HbA1c has been associated with a 10% reduction in diabetes-related death and 25% reduction in microvascular complications, with greater reductions when simultaneously targeting glucose, blood pressure and lipid control^44,45^. These could lead to a reduction in direct healthcare cost (through fewer visits to healthcare professionals, reduced absence from work and hospital admissions) as well as positive impacts on patients’ quality of life^46^.

Our findings were less clear in several areas. Due to the limited number of studies included in each subgroup, it was not possible to clearly identify whether different technology used to support telemedicine (e.g. computer, SMS, smartphones), use of interactive or non-interactive technology, administrator of intervention (e.g. nurse, physician) or a combination of these factors had led to improved results. We were also unable to assess these interactions in our meta-regression analyses due to the small number of trials for each outcomes as well as the substantial heterogeneity of results. Most of the trials included had only reported HbA1c concentrations, with very few reporting other key aspects of diabetes managements. As such, we urge caution when interpreting results for other secondary outcomes, though our preliminary analyses suggest some consistency across outcomes. The evidences presented in this review are in agreement with other systematic reviews which suggest that telemedicine is useful in improving patient care (Supplemental Table S11), with similar magnitude of improvements. The present review also showed that telemedicine strategies were associated with additional clinical benefits beyond glycaemic control and these included improvement in blood pressure and triglyceride levels.

In our review, we also found that the definition of usual care was inconsistent across all studies. These varied from regular clinic visit every 3 to 6 months to a more intensive form of usual care, including drug therapy review, dietary counseling and health coaching. While we attempted to stratify the various elements that constituted to usual care, the descriptions provided by most studies were not sufficiently detailed. As such, it is possible that the relative effectiveness of telemedicine noted in this study maybe underestimated, especially if implemented in routine clinical practice.

In the present review, a high level of clinical heterogeneity of studies was found and this was probably due to the diverse patient population recruited. Such variation is expected for an analysis of complex intervention and hence, a meta-regression approach was utilized in the present study. This study found that study location was a significant predictor for larger reductions in HbA1c, explaining 31% of the heterogeneity. This could be attributed to several reasons. Firstly, the different settings where these studies were conducted could mean that some of the contextual (such as health care system) and cultural factors may have played a role in the implementation process and thus affected the outcomes^30,47^. In addition, most of the studies conducted in Asian countries had smaller sample sizes and were conducted over a shorter period of time. This might have increased adherence to the intervention and hence a better success rate. Similarly, the implementation of intervention could also be more intense in studies with smaller sample size. Lastly, there could be variation in the dissemination and implementation dimensions of such intervention between the continents.

The present study has several important limitations that warrant mentioning. Firstly, the high level of heterogeneity in the results suggests that the results should be interpreted with caution. This could be attributed to the variation in types of telemedicine technology used, population studied as well as healthcare personals involved. Secondly, as with all meta-regression analyses, we used summary data which made the findings vulnerable to ecological fallacy, and thus these findings may not be applicable to an individual. In addition, there is limited statistical power with these analyses^48^. Thirdly, the lack of long term follow-up (minimum 1 to 2 years) studies may limit the interpretation of the results to only short-term effects of included interventions and not long-term outcomes such as mortality. The sustainability and cost-effectiveness of these interventions may also be questionable. Most of the results used in the meta-analyses were based upon per protocol analysis, which may have resulted in higher result estimates. Due to the complexity of interventions involved, compounded by a lack of description in most studies, misclassified interventions could not be ruled out. To minimize the misclassification of interventions, two authors independently assessed each study and reviewed other pertaining report of the same study to obtain additional information. This technique has been found to improve reporting completeness by nearly 65%^49^. The lack of reporting for many trials had also limited our ability to perform several subgroup analyses such as roles of interventionist and healthcare setting on the impact of telemedicine. Finally, only a few studies had provided data on adverse events, quality-of-life measures or even cost-effectiveness analyses and hence, the data should be interpreted with caution.

Investments in information technology will increase over the next few years. The network meta-analysis in the present study substantiates that individual focused telemedicine intervention such as education, small group discussion as well as structural changes (replacing clinic visits with remote consultations) is effective in reducing HbA1c. Additionally, it provides some important insights for future research development and implementation. This includes a more detailed description of the methodology as well as target other clinical outcomes besides HbA1c, such as quality of life and cost savings. Such information is vital for policy-makers to tailor their choice of interventions to the desired outcomes based on the best available evidence, maximizing available resources in a local healthcare context. The challenge, however, remains as to how to ensure such evidence does not get lost in translation and can be adopted within reasonable timeframe. Some strategies may include the promotion of access and usage by practitioners through capacity building, dissemination of intervention materials and engaging stakeholders in the planning phase.

## Conclusion

The greatest potential of telemedicine lies in providing easy access especially by people in rural areas where healthcare resources are limited. Assessing the acceptability and implementation challenges of telemedicine in resource poor areas is an important next step to accelerate translation. This can lead to reduced healthcare cost and improved patient outcomes.

## Electronic supplementary material

Supplementary Information
