# Supplementary material for: Comparative effectiveness of telemedicine strategies on type 2 diabetes management: A systematic review and network meta-analysis
Source: Sci Rep. 2017 Oct 4;7:12680. doi: 10.1038/s41598-017-12987-z (PMC5627243; doi:10.1038/s41598-017-12987-z)
Supplement: Supplementary file 1 — Supplementary Information [file 41598_2017_12987_MOESM1_ESM.pdf]

Comparative effectiveness of telemedicine strategies on type 2 diabetes management: A systematic review and network meta-analysis

Shaun Wen Huey Lee, Carina Ka Yee Chan, Siew Siang Chua, Nathorn Chaiyakunapruk

Supplementary Online Content

Table S1 Literature search strategy
Table S2 Description of interventions and outcomes
Table S3 Selected baseline demographics and details of intervention strategies employed
Table S4 Key intervention component of studies included in the current review
Table S5 Details of educational content delivered to participants in studies examining teleeducation
Table S6 Details of self-monitoring frequency and recommended schedule
Table S7 Details of the action(s) taken based upon results from glucose monitoring
Table S8 Results of subgroup and sensitivity analysis for primary outcome
Table S9 Results of the network meta-analyses
Table S10 Inconsistency estimates
Table S11 Summary of effect sizes comparing meta-analysis from other studies and current study

Figure S1 Flow chart depicts the process of study selection
Figure S2 Risk of bias assessment appraisal of randomized controlled studies on telemedicine using the Cochrane risk of bias tool
Figure S3 Evidence network for outcomes assessed
Figure S4 Surface under curve ranking
Figure S5 Comparison adjusted funnel plot
eReferences List of studies included in the current review

Table S1 Literature search strategy

We searched MEDLINE using the algorithm listed below. The algorithm was adjusted to also search the Cochrane Library,EMBASE, PsycINFO, Web of Science and CINAHL databases. This was supplemented by a search of grey literature in International Pharmaceutical Abstracts, Australasian Digital Theses Program, Aslib Index to Theses and ProQuest Digital Dissertation, and Theses.

Database: Ovid MEDLINE® <1970 to September Week 4 2016> Search Strategy:

1. exp diabetes mellitus, type 2
2. NIDDM.mp
3. T2DM.mp
4. non insulin* depend*.mp
5. noninsulin* depend*.mp
6. non insulin depend*.mp
7. noninsulin depend*.mp
8. #1 OR #2 OR #3 OR #4 OR #5 OR #6 OR #7
9. Telehealth.mp
10. telemedicine.mp
11. telemonitoring.mp
12. telecare.mp
13. telematics.mp
14. telehomecare.mp
15. mobile health.mp
16. remote monitoring.mp
17. #9 OR #10 OR #11 OR #12 OR #13 OR #14 OR #15 OR #16
18. remote consultation.mp
19. sms.mp
20. short messaging system.mp
21. video monitoring.mp
22. internet monitoring.mp
23. internet consultation.mp
24. video consultation.mp
25. telephone.mp
26. smartphone.mp
27. cellular phone.mp
28. mobile phone.mp
29. telephone support.mp
30. #18 OR #19 OR #20 OR #21 OR #22 OR #23 OR #24 OR #25 OR #26 OR #27 OR #28 OR #29
31. Randomized controlled trial.mp
32. randomized controlled study.mp
33. clinical trials.mp
34. prospective*.mp
35. #31 OR #32 OR #33 OR #34
36. #8 AND #17
37. #30 AND #36
38. #35 AND #37

Table S2 Description of content of interventions and outcomes

Studies were coded to determine the strategies used in the intervention and control groups. These are broadly categorized as follow:-

1. Individualized care plan – Use of a specific structured algorithm to identify and individualize treatment / care to the patient
2. Audit & Feedback – Evidence of providing feedback based upon patient reported results. Feedback provided to the patient can include communication (both, 1-way or 2-way) or shared decision making strategies between patient and provider to implement plan
3. Psychology based counselling – Any intervention which aims to modify behavior, belief and perceptions of the patient
4. Skill building - Use of any intervention that explicitly aims to educate and develop skills which focuses to promote a change in behavioral domains relevant to diabetes
5. Financial incentive – Use of incentives to patient as a motivation to improve diabetes care (eg. attendance at scheduled visits, compliance to medications, meeting target glycaemic goals)
6. Peer / Family support – Intervention which involves someone / family member with diabetes who is adherent to treatment and lifestyle guideline, to provide support, set goals or formulate plans to maintain self-care behaviors over time.
7. Theory based counselling – Any educational intervention (including self-management, self-monitoring of blood glucose and/or behavior change) described for participants.
8. Lay health advisor – An intervention which trains community members to target diabetes patients and in some cases, known and trusted members of the community.

Table S3: Selected baseline demographics and details of intervention strategies employed

Author, Year (Country)	Comparison group	Key elements of intervention	Sample size, n	Age, yrs	Men, No (%)	Medium used for intervention	Frequency of intervention and duration	Intervention team	Psychological model used in intervention
**Articles examining teleeducation**									
Arora, 2014 (USA)	Usual care	Two daily educational text message over 6 months on promoting health behaviour	128	51	83 (65)	SMS	2 text messages daily; 6 months	NR	Educational, Behavioural
Avdal, 2011 (Turkey)	Basic diabetes education in polyclinic	Web-based diabetes education	122	52	60 (49)	Internet	NR	DE	Educational, Behavioural
Bell, 2012 (USA)	Usual care	30-60 seconds video message sent daily to mobile phone over 6 months on self- efficacy management and education topics outline by American Association of Diabetes Educators	64	58	35 (55)	Mobile phone	30-60 seconds video message daily; 6 months	RN	Educational, Behavioural, Cognitive, Psychosocial
Blackberry, 2013 (Australia)	Standard diabetes care including referrals to diabetic educators, dieticians and diabetes specialists	Nine structured patient coaching on diabetes self-management including lifestyle issues, medication adherence and dosing and self-monitoring	473	63	270 (57)	Telephone	9 sessions; 15 months	RN	Educational, Behavioural
Bond, 2007 (USA)	Usual care	Weekly messages on behavioral and motivational strategies	62	NR	34 (55)	Internet	Weekly; 6 months	RN	Educational, Behavioural, Cognitive

		and cues to improve diabetes self-management and change personal behavior							
Browning, 2016 (China)	Usual medical care but medical fees payment waived	Coach led motivational health coaching. Higher intensity at the beginning of study	711	64	336 (47)	Telephone	4 calls; 12 months	RN	Educational, Cognitive
Capozza, 2015 (USA)	Usual care	Daily education messages (1-7 messages) related to diabetes and health improvement	93	50	34 (37)	SMS	Daily; 6 months	NR	Educational
Davis, 2010 (USA)	Usual care with one 20-minute education session	Interactive patient education comprising on 13 modules on self-management support	165	60	42 (26)	VC, Telephone	13 sessions; 12 months	DE, Dietician	Educational, Behavioural
Glasgow, 1997 (USA)	Usual care with quarterly follow-up on microvasular and macrovascular risk factor assessment	Computer generated individualized goal setting plan with follow up. Patients with high self-efficacy levels received a self-review video while those with lower self-efficacy had a 30 minute interactive video and telephone follow-up	206	62	79 (38)	Computer	2 telephone calls; 6 months	NR	Educational, Behavioural
Glasgow, 2006 (USA)	Usual care by physician supplemented with a generic health-risk appraisal	Computer assisted patient education focusing on healthy eating and physical activity with a tailored follow up letter	335	62	168 (50)	Computer	2 sessions; 1 month	Peer support	Educational, Behavioural, Cognitive, Psychosocial

Goodarzi, 2012 (Iran)	Usual care	Four educational messages weekly focusing on diet, exercise and medications delivered by SMS over 12 weeks	81	54	18 (22)	SMS	48 messages; 3 months	NR	Educational, Psychosocial
Graziano, 2009 (USA)	Usual care which include clinic visit every 2-3 months	Daily automated pre-recorded voice-message over 3 months focusing on behaviors, attitude, beliefs and reduction of barriers related to performing self-care behavior.	119	62	66 (55)	Telephone	Daily; 3 months	DE	Financial, Educational, Behavioural
Islam, 2015 (USA)	Usual care	90 SMS educational messages for 6 months based upon behavioural learning theory and transtheoretical model of behavioural change	236	48	108 (46)	SMS	6 months	NR	Educational, Behavioural
Kim, 2003 (Korea)	Usual care	16 telephone calls over 12 weeks educating patient and reinforcement of lifestyle changes	36	60	11 (31)	Telephone	16 calls; 3 months	RN, Dietician	Educational, Behavioural
Lee, 2007 (Taiwan)	Usual care supplemented with patient education	An integrated web-based patient information system which can deliver patient educational messages on disease management and skill-building	274	58	141 (51)	Internet	NR	NR	Educational, Behavioural
Leu, 2005 (USA)	Usual care	Automated system which delivers health-related messages (dietary, exercise, meal-time reinforcements) as well as	50	NR	NR	Pager	NR	NR	Educational

		medication and appointment reminders							
Liou, 2014 (Taiwan)	Usual care with one individual diabetes education session	Six monthly session on and patient education on diet, medication, stress management, goal setting and foot care	95	57	48 (51)	VC	6 sessions; 6 months	NR	Educational
Lorig, 2008 (USA)	Usual care	Fifteen automated telephone delivered diabetes self- management education (90 second vignette each)	417	53	159 (38)	Telephone	15 sessions; 18 months	Peer	Educational, Behavioural
McKay, 2002 (USA)	Usual care with information provided via internet	Twice weekly personalized diabetes self-management coaching over 3 months to reach dietary goal	160	60	75 (47)	Internet	Up to 2 contacts per week; 3 months	None	Educational, Behavioural
		Online peer support system to exchange diabetes-related information, coping strategies and emotional support				Internet	Live chat forum; 3 months	Peer	Educational, Behavioural, Psychosocial
		Combination of diabetes self-management and peer support				Internet	Up to 2 contacts per week with live chat forum; 3 months	Peer	Educational, Behavioural, Cognitive, Psychosocial
Noh, 2010 (Korea)	Usual care	Web-based education on diet, hypoglycaemic symptoms, diabetes and stress management	40	42	31 (78)	Internet	Every two months	NR	Educational

Peimani, 2016	Usual care	General educational message on diabetes self-care	150	53	81 (54)	SMS	7 messages a week; 3 months	NR	Educational, Cognitive
		Tailored educational messages on adherence and self-care based upon barriers identified				SMS	7 messages a week; 3 months		Educational, Cognitive
Piette, 2011 (USA)	Usual care	12-weekly session with 9 monthly booster session of cognitive behavioural therapy module delivered by telephone focusing on depressive symptoms, self-management and physical activities	291	56	144 (50)	Telephone	21 sessions; 12 months	RN	Educational, Behavioural, Cognitive, Psychosocial
Plotnikoff, 2013 (Canada)	Usual care with standard print educational materials	Patient education (print materials) with pedometer	287	62	155 (54)	NA	Mailed print material every 3 months; 12 months	NR	Educational,
		Patient education with print materials, pedometer and counselling				Telephone	16 calls; 12 months	NR	Educational, Cognitive, Psychosocial
Sacco, 2009 (USA)	Usual care	Regular telephone delivered coaching and education on self-management by paraprofessionals	62	NR	NR	Telephone	18 sessions; 6 months	Peer	Educational, Behavioural, Cognitive, Psychosocial
Shetty, 2011 (India)	Standard care including appropriate drug therapy, laboratory investigations	Reminder once every 3 days on dietary modification, physical activity and drug schedules	215	50	NR	SMS	Every 3 days for 12 months with clinic visits every 3 months	NR	Educational, Behavioural

	and advice on diet modification and physical activity								
Whittemore, 2004 (USA)	Usual care	Five individualized session and 2 follow-up calls by nurses over 6 months. Coaching session involve educational, behavioral, and affective strategies.	49	NR	NR	Telephone	8 sessions; 6 months	RN	Educational, Behavioural, Cognitive
Williams, 2012 (Australia)	Usual care with quarterly newsletter on health information	Weekly automated interactive telephone call system to improve self-management behaviours over 6 months. Each call last between 5-20 minutes.	120	57	75 (63)	Telephone	Weekly; 6 months	NR	Educational, Behavioural
Wolever, 2010 (USA)	Usual care	Integrated health coaching on psychosocial, behavioural and glycaemic control 14 times over 6 months. Each session lasts 30 minutes.	56	53	13 (23)	Telephone	13 telephone calls; 6 months	Psychologist	Educational, Behavioural, Cognitive, Psychosocial
Yoon, 2008 (Korea)	Usual care supplemented with patient education during clinic visits	Educational messages sent by SMS to patients on self-monitoring and medications weekly. Patients could also review information electronically and receive recommendations online.	51	47	22 (43)	SMS	52 times; 12 months	RN, DE, Endocrinologist	Educational, Behavioural, Psychosocial

Zolfaghari, 2011 (Iran)	Four educational messages weekly covering topics such as diet, exercise, medication and glucose monitoring over 12 weeks.	Patient self-efficacy education and counselling by telephone on similar topics in SMS group. Frequency was twice a week for first month, then weekly for months 2 & 3.	77	52	36 (47)	Telephone	16 calls; 3 months	NR	Educational, Behavioural, Psychosocial
**Articles examining telecase-management**									
Anderson, 2011 (USA)	Usual care	Semi-structured telephone disease management between clinic visits. Calls were weekly, biweekly or monthly and focused on clinical assessments, self-management, adherence and glucose monitoring.	295	NR	124 (42)	Telephone	Variable depending on HbA1c levels; 12 months	RN	Educational, Behavioural
Aubert, 1998 (USA)	Usual care with diabetes education	Coordinated nurse care management of diabetes based on 6 step algorithm to improve glycemic control through medication adjustment and lifestyle changes. Nurse case manager met with family medicine physician and endocrinologist biweekly to review patient progress and other issues.	138	NR	55 (40)	Telephone	Biweekly telephone calls; 12 months	RN. DE	Educational, Behavioural, Cognitive
Glasgow, 2010	Enhanced usual care	Computer assisted web-based self-management (CASM) which	463	58	232 (50)	Computer	Weekly feedback; 4 months	NR	Educational, Behavioural

(USA)		participants selected treatment goals and monitored progress. Participants received feedback and periodic motivational calls over 12 weeks.							
		Computer assisted self-management (CASM) with two calls from interventionist to support participants.				Computer, Telephone	Weekly feedback and 2 telephone follow up; 4 months	Social worker	Educational, Behavioural
Huizinga, 2010 (USA)	Usual care	Patient case management by nurse with referral to dietician if necessary. Interventionist helped participants titrate medication, identify and solve self-care behaviours such as diet, exercise and self-monitoring.	164	55	92 (56)	Telephone	Monthly or quarterly ; 24 months	RN	Educational, Behavioural
Hussien, 2011 (Bahrain)	Usual care and physician visit	Interaction with patient via SMS on problem solving, skill building, advice and action plans in between clinic visits. Reminder SMS sent every 7 days to non-respondents	34	47	NR	SMS	Weekly; 3 months	Physician	Educational, Behavioural, Psychosocial
Piette, 2000 (USA)	Usual care	Automated telephone disease management (ATDM) which consists of automated assessment and structured educational messages on self-care	248	55	102 (41)	Telephone	Weekly; 6 months	RN	Educational, Behavioural

		delivered bi-weekly. Nurse educators called participants to addressed issues identified weekly.							
Ralston, 2009 (USA)	Usual care	Diabetes web-based care-management program by case manager which included patient electronic medical record access, feedback via emails, educational website and interactive diary.	83	57	42 (51)	Internet	Minimum weekly; 12 months	RN	Educational, Behavioural
Shea, 2009 (USA)	Usual care	Web-based patient case management based upon clinical guidelines	1665	71	619 (37)	Internet, VC	NR; 12 months	RN	Educational, Behavioural
Wayne, 2015 (Canada)	Health coaching to achieve health-behaviour changes	Health coaching with live-support by mobile phone	131	54	27 (28)	Mobile phone	Daily; 6 months	Health coach	Educational, Behavioural
Zhou, 2014 (China)	Usual care and educational session for diabetes self-management and nutrition	Patient educational message on self-management with monitoring	108	NR	NR	Internet, Telephone or SMS	Every 2 weeks; 3 months	NR	Educational, Behavioural, Psychosocial
**Articles examining telemonitoring**									
Boaz, 2009 (Israel)	Usual care	Remote patient monitoring with feedback and advice from nurse in the event blood values exceed range of 50-280mg/dL.	35	63	13 (37)	Internet	NR	DE	Behavioural

Bujnowska-Fedak, 2011 (Poland)	Usual care	Remote patient monitoring at home with feedback in cases that require urgent medical attention (hypoglycemia)	95	55	51 (53)	Internet	NR; 6 months	RN	Behavioural
Cho, 2006 (Korea)	Usual care supplemented with diabetes education	Remote patient monitoring with feedback by clinical instructors every 2 weeks	80	53	49 (61)	Internet	Every fortnight; 30 months	Endocrinologist, RN, Dietician	Educational, Behavioural
Cho, 2009 (Korea)	Internet based remote patient monitoring	Remote patient monitoring via mobile phone	69	48	54 (78)	Mobile phone	Every fortnight; NR	RN, Dietician	Educational, Behavioural
Earle, 2010 (UK)	Usual care	Remote patient monitoring of blood glucose and blood pressure. Data recordings uploaded weekly.	137	58	NR	Mobile phone	Once a week; 6 months	GP	Educational, Behavioural
Kim, 2016 (China)	Usual care with baseline education	Remote patient monitoring with feedback weekly during 1st 3 months then biweekly thereafter.	182	54	88 (48)	Internet	At least once weekly; 6 months	RN	Behavioural
Kwon, 2004 (Korea)	Usual care with monthly clinic visit	Remote patient monitoring for 12 weeks	110	54	67 (61)	Internet	At least once weekly; 3 months	RN, Dietician	Educational, Behavioural
Lee, 2015 (Malaysia)	Usual care supplemented with Ramadan focused education session	Remote patient monitoring with feedback from interventionist	37	51	16 (43)	Internet	5 times daily for 4 weeks	Pharmacist	Behavioural

Lim, 2011 (Korea)	Usual care with one diabetes education session	Self-monitoring of blood glucose (SMBG) at least 8 times weekly	154	68	64 (42)	Internet	NR	NR	Behavioural
		Remote patient monitoring as per SMBG group with feedback				Mobile phone	Weekly; 6 months	NR	Educational, Behavioural
Luley, 2011 (Germany)	Usual care	Integrated program comprising of remote patient monitoring with dietary changes	70	NR	NR	Internet	Weekly; 6 months	NR	Educational, Behavioural, Psychosocial
Nicolucci, 2015 (Italy)	Usual care	Home telehealth system which transmits data real-time to a central system. Nurses contact patient monthly by phone to discuss results and identify barriers to compliance	302	58	186 (62)	Internet, SMS, telephone	Twice a month; 12 months	Physician, RN	Educational, Behavioural
Or, 2016 (Hong Kong)	Usual care with self-monitoring of blood glucose	Patient-centered, computer based remote patient monitoring with feedback	63	70	20 (32)	Computer	NR	NR	Behavioural
Pressman, 2014 (USA)	Usual care	Remote patient monitoring with online health diary. Measurements sent weekly with care managers responding based upon target goals.	225	56	139 (62)	Internet	Weekly; 6 months	Physician, DE	Educational, Behavioural
Quinn, 2011 (USA)	Usual care with SMBG	Home telemonitoring with feedback	163	53	81 (50)	Mobile phone		RN	Behavioural

		Home telemonitoring with feedback and access by physician to unanalyzed data					Every 2.5 months; 12 months	RN, Physician	Educational, Behavioural
		Home telemonitoring with feedback and access by physician to patient summary data and treatment recommendation						RN, Physician	Educational, Behavioural
Tildesley, 2011 (Canada)	Usual care with option of calling endocrinologist for assistance when required	Remote patient monitoring on glucose every 2 weeks with feedback from endocrinologists	47	59	29 (62)	Internet	Data upload every 2 weeks	Endocrinologist	Educational, Behavioural
Yoo, 2009 (Korea)	Usual care	Remote monitoring, online exercise diary and patient reminder	111	58	65 (59)	Mobile phone	3 SMS per day; 3months	RN	Educational, Behavioural
**Articles examining teleconsultation**									
Ciemins, 2011 (USA)	Multidisciplinary team diabetes care management using face-to-face visit	Multidisciplinary team diabetes care management in collaboration with local PCP via teleconferencing	206	62	80 (39)	VC	NR	DE, Dietician, Physician	Educational, Behavioural
Leichter, 2013 (USA)	Usual care with quarterly clinic visit	Replacement of clinic visits at month 3 & 9 with remote consultation	98	48	55 (56)	Internet, Telephone	Every 3 months	Endocrinologist	Educational, Behavioural
Nagrebetsky, 2013 (UK)	Usual care with supportive lifestyle-focused education	Stepwise diabetes treatment program delivered remotely with supportive lifestyle-focused education	14	58	10 (71)	Telephone	Twice weekly; 6 months	RN	Educational, Behavioural

Rasmussen, 2015 (Denmark)	Usual care	Home treatment by physicians via video consultation	40	NR	27 (67)	Internet	Every 3 weeks; 6 months	Physician	Behavioural
Shahid, 2015 (Pakistan)	Usual care and physical examination after 4 months	Remote patient consultation and feedback on glucose monitoring, medications and lifestyle every 15 days for 4 months	440	49	270 (61)	Mobile phone	Every 15 days; 4 months	RN	Educational, Behavioural
Smith, 2008 (USA)	Usual care	Patient teleconsultation by endocrinologist	635	NR	300 (47)	Internet	Unclear	Endocrinologist	Educational
Whitlock, 2000 (USA)	Routine usual care	Weekly visits by case manager and monthly visit by physician using home telemedicine unit	28	63	11 (39)	Internet	Weekly and monthly; 3 months	RN, Physician	Behavioural
Wong, 2005 (Hong Kong)	Routine hospital care	Remote patient management based upon patient health behaviour	101	62	57 (56)	Telephone	Every 1-2 weeks; 6 months	RN	Educational, Behavioural
**Articles examining telementoring**									
Anderson-Loftin, 2005 (USA)	Usual care with referral to diabetes class	Culturally appropriate educational classes on dietary strategies, peer-professional group discussion and telephone follow up	97	57	21 (22)	Telephone	Weekly; 6 months	Dietician, DE, Peer	Educational, Behavioural, Psychosocial
Chan, 2014 (Hong Kong)	Integrated care implemented through a web-based multi-component quality	Integrated care implemented through a web-based multi-component quality improvement program with telephone based support	628	55	355 (57)	Internet & Telephone	Biweekly for first 3 months, then once every other month for 6 months; 12 months	Physician, RN, Peer	Educational, Behavioural, Psychosocial

	improvement program								
Dale, 2009 (UK)	Usual care	Peer or nurse support delivered by telephone to motivate adherence and enhance self-efficacy among patients. Calls delivered 6 times at days 3-5, 7-10, 14-18, 28-35, 56-70 and 120-150.	231	NR	135 (58)	Telephone	6 sessions; 6 months	RN, Peer	Behavioural, Psychosocial
Dang, 2013 (Vietnam)	Usual care supplemented with diabetes self-care booklets	Four classroom education by research team on diabetes self-education, monitoring, diet and stress management. Weekly call by peer leader for 1st 2 months and biweekly for the next 3 months	102	NR	NR	Telephone	Weekly for first 2 months and biweekly every 3 months; 6 months	Peer	Educational, Behavioural, Psychosocial
Heisler, 2010 (USA)	Enhanced usual care with nurse care management	Paired peer with weekly telephone contact and optional group sharing	244	62	244 (100)	Telephone	Minimum once weekly; 6 months	RN, Peer	Educational, Behavioural, Psychosocial
Keyserling, 2002 (USA)	Usual care	Face to face clinic education with peer counsellor mentoring	200	59	NR	Telephone	Four clinic education session and monthly telephone calls with 3 group sessions; 12 months	Physician, DE, Peer	Educational, Behavioural, Cognitive, Psychosocial
		Face to face clinic education peer					4 clinic education session only	Physician, DE	

Long, 2012 (USA)	Usual care	Matched peer mentor to help identify the differences in behavior and goals and help identify realistic plans for goal achievement	117	60	110 (94)	Telephone	Minimum once weekly; 6 months	Peer	Educational, Behavioural, Psychosocial
		Financial incentive based upon HbA1c goals achieved				N/A	N/A	N/A	Financial
Samuel-Hodges, 2009 (USA)	Usual care with pamphlets on diabetes self-care	Individual dietary assessment, goal setting, education and telephone contact by church diabetes advisors with 2 printed message from physician	201	59	73 (33)	Telephone	Twelve bi-weekly group session with monthly telephone follow up by church diabetes advisors; 6 months	Physician, Pharmacist, RN, Dietician, Peer	Educational, Behavioural, Cognitive, Psychosocial
Spenser, 2011 (USA)	Usual care with access to community activities such as healthy eating demonstration, physical activity and weekly community farmers' produce	Usual care with access to family health advocates which promoted healthy lifestyle and diabetes self-management activities through education, two home visits, one clinic visit with doctor with advocate and telephone calls every 2 weeks.	111	59	23 (21)	Telephone	Telephone calls every 2 weeks; 6 months	Peer	Educational, Behavioural, Psychosocial
Thom, 2013 (USA)	Usual care including access to nutritionist and diabetes educator through referral from primary care provider	Paired peer coach with telephone call at least twice monthly, minimum two face to face visits and one clinic visit if possible	299	55	143 (48)	Telephone	Telephone calls every 2 weeks; 6 months	Peer	Educational, Behavioural, Psychosocial

Walker, 2011 (USA)	Usual care and provision of self-management materials	Up to 10 calls at 4 to 6 weeks intervals by health educator over 1 year. Calls focused primary on patient empowerment to improve self-efficacy, medication adherence and lifestyle changes	526	56	173 (33)	Telephone	Up to 10 calls; 12 months	Health educator	Educational, Behavioural
**Articles examining teleeducation & telecase-management**									
Carter, 2011 (USA)	Usual care	Integrated care with self-management, education and nurse support delivered by VC. Action plans developed bi-weekly during the 30 min conferences with patients.	47	49	17 (36)	VC	Biweekly ; 9 months	RN	Educational, Behavioural, Cognitive
Cho, 2011 (Korea)	Usual care with SMBG and diabetes education	Remote patient coaching and management by physician. Face-to-face education by nurses.	71	64	28 (39)	Internet	Weekly; 3 months	RN, Physician	Educational, Behavioural
Crowley, 2013 (USA)	Usual care	Standardised educational modules delivered by nurse to enable patient self-management and behavioural modification. Interventionist also facilitate medication management by providing summaries to attending physicians who will make the decision on medication changes	359	57	101 (28)	Telephone	Monthly telephone call over 12 months. Additional contact to doctor every 3 months on medication management	NR	Educational, Behavioural, Cognitive

Holmen, 2014 (Norway)	Usual care	Mobile based application to increase self-management through education	151	57	89 (59)	Mobile phone	Daily; 1 year	NR	Educational, Behavioural
		Mobile based application to increase self-management through education with motivational and health counseling. Structured counseling over 4 months (5 sessions) by nurse.				Mobile phone	Daily; 1 year, 5 education sessions; 4 months	RN, dietician	Educational, Behavioural, Cognitive
Maljanian, 2005 (USA)	Usual care supplemented with diabetes education and collaborative care management	As per control group with additional weekly calls to reinforce patient education and self-management skills over 12 weeks. Calls were focused to reinforce importance of glycemic control, complications and comorbidities.	336	58	157 (47)	Telephone	12 sessions; 12 months	RN, Nutritionist	Educational
Nesari, 2010 (Iran)	Usual care supplemented with diabetes education	Reinforcement of diabetes self- management, education and drug adjustment. Telephone calls were at least twice weekly for 1 month and weekly for next 2 months	60	51	17 (28)	Telephone	16 calls; 3 months	NR	Educational, Behavioural
Oh , 2003 (Korea)	Usual care	Continuous patient education, lifestyle adjustment advice with drug adjustment. Telephone calls were at least twice weekly for 1	50	61	18 (36)	Telephone	Twice a week for 1st month, once a week for 2nd and 3rd months	NR	Educational, Behavioural

		month and weekly for next 2 months.							
Piette, 2001 (USA)	Usual care	Automated structured educational messages. Weekly nurse follow-up based upon participants response to educational messages and discussed appropriate self-care issues.	272	61	264 (97)	Telephone	Weekly; 12 months	RN	Educational, Behavioural
Schillinger, 2009	Usual care	Automated calls to support patient in diabetes self-care management and psychological issues in patients' native language as well as monitoring and health educational messages. Case-manager assist patient to problem solve and individualise action plans	339	55	139 (41)	SMS	Weekly over 39 weeks	Nurse	Educational, Behavioural, Cognitive
		Monthly group medical visits to primary healthcare physician and health educator				Face-to-face	Monthly for 9 months; 90 minutes each session	Physician, Pharmacist	Educational, Behavioural, Financial
Tjam, 2006 (Canada)	Usual care	Interactive online disease management program and patient education	57	NR	NR	Internet	NR	RN	Educational, Behavioural
Young, 2005 (UK)	Usual care	Structured telephone calls to support and guide patients on self-efficacy. Frequency of calls varied,	591	NR	343 (58)	Telephone	Variable depending on HbA1c levels; 12 months	RN, Peer	Educational, Behavioural, Cognitive, Psychosocial

		depending on last HbA1c level.							
**Articles examining teleeducation & telemonitoring**									
Chen, 2011 (Taiwan)	Usual care	Intensive diabetes educational program with remote patient monitoring	64	54	29 (46)	Internet	6 sessions; NR	DE	Educational, Behavioural
Faridi, 2008 (USA)	Usual care and use of pedometer	Tailored patient educational messages with remote monitoring and use of pedometer	30	56	11 (37)	Mobile phone	Daily; 3 months	NR	Educational, Behavioural
Greenwood, 2015 (USA)	Usual care with diabetes education as needed	Sequential daily health sessions and structured remote monitoring	90	56	69 (77)	Internet	Daily; 6 months	DE, Physician	Educational, Behavioural
Karhula, 2015	Usual care and disease management educational booklet	Health coaching every 4-6 weeks (30 mins each) and remote patient monitoring (weekly)	250	66.2 (8.6)	139 (56)	Internet & mobile phone		Health coach	Educational, Behavioural
Lim, 2016 (Korea)	Usual care with baseline education and self-monitoring of blood glucose	Individualized multidisciplinary U-healthcare system with feedback	100	65	75 (75)	Mobile phone	Daily; 6 months	NR	Behavioural,
Steventon, 2015 (UK)	Usual care	Remote telemonitoring with feedback via video, message to educate patients on their conditions	513	65	NR	Internet	Variable; 12 months	RN	Educational, Behavioural, Psychosocial

Tang, 2013 (USA)	Usual care	Personalized video educational message and remote monitoring	415	54	249 (60)	Internet	3 in person visit and tailored online monitoring; 6 months	RN, DE, Pharmacist, Dietician	Educational, Behavioural
Wakefield, 2011 (USA)	Usual care	Remote patient monitoring	302	68	296 (98)	Telephone	Weekly; 12 months	RN, DE, Physician	Behavioural, Psychosocial
		Remote patient monitoring with prompts and educational messages on disease management							Educational, Behavioural, Psychosocial
**Articles examining telecase-management & telemonitoring**									
Anzaldo-Campos, 2016 (Brazil)	Usual care	Combined care management and peer support	301	52	100 (33)	Face-to-face	8 session with additional non-compulsory sessions; 10 months	RN, Peer	Behavioural, Psychosocial
		Combined care management, peer support with remote patient monitoring				Internet	Variable but intensity decreases over time;	RN, Peer	Behavioural, Psychosocial
Castelnuovo 2011, (Italy)	Hospital-based treatment and usual follow-up when necessary	In-patient nutritional & psychological counselling with web-based monitoring and counselling	34	-	18 (53)	Internet	NR	DE	Educational, Behavioural
Del Prato, 2012 (Italy)	Usual SMBG	Remote patient monitoring of blood glucose and self-management	241	58	125 (52)	Handheld device	Daily; 6 months	Endocrinologist	Behavioural, Psychosocial

Kim, 2008 (Korea)	Usual care	Daily patient monitoring of glucose and disease management sent by SMS or internet weekly	34	47	16 (47)	SMS	Weekly; 12 months	DE, RN	Educational, Behavioural
Kim, 2009 (Korea)	Delayed intervention	Integrated diabetic self-help, management and remote glucose monitoring. Structured counseling based upon average monthly results.	79	56	44 (56)	Internet, Telephone	6 education session & 6 telephone call; 6 months	RN	Educational, Behavioural
McMahon, 2005 (USA)	Usual care supplemented with diabetes education	Web-based care management & monitoring	104	64	103 (99)	Internet	NR; 12 months	DE, Physician	Educational, Behavioural
McMahon, 2012 (USA)	Usual care with Internet training	Online diabetes care management with monitoring	151	60	143 (95)	Internet	NR	RN, Pharmacist	Educational, Behavioural
		Diabetes self-management with monitoring				Telephone	NR	RN, Pharmacist	Educational, Behavioural
Orsama, 2013 (Finland)	Usual care	Remote patient monitoring and reporting of health related parameters. Automated personalized feedback sent based upon reported measurements	48	62	26 (54)	Mobile phone	Weekly; 10 months	Physician, RN	Educational, Behavioural
Rodriguez-Idigoras, 2009 (Spain)	Usual care	Remote patient monitoring with telephone based-care and consultation	328	NR	169 (52)	Mobile phone	NR; 12 months	Physician, RN	Behavioural

Stone, 2010 (USA)	Monthly care coordination	Active care management and remote home monitoring	137	NR	NR	Internet	Daily transmission; 6 months	RN	Educational, Behavioural
Stone, 2012 (USA)	Usual care	Active patient care only	101	NR	99 (98)	Telephone	Monthly; 6 months	RN	Educational
	Care coordination	Active care management and remote home monitoring				Telephone, Internet	Daily; 6 months	RN	Educational, Behavioural
Waki, 2014 (Japan)	Usual care	Remote patient monitoring and self-management	54	55	41 (76)	Internet	Variable; 3 months	Physician, RN	Behavioural
**Articles examining telecase-management & teleconsultation**									
Hsu 2016, (USA)	Education on insulin injection based upon standardised protocol	Remote patient monitoring with feedback	40	54	-	Internet	NR	DE	Educational, Behavioural

NR- Not reported, SMS – Short messaging system, RN – Registered nurse, DE- Diabetes educator

Table S4: Key intervention component of studies included in the current review

Author		Intervention									Control								
		Individualised care plan	Audit & Feedback	Psychology based counselling	Skill building	Financial incentive	Problem solving	Peer / Family support	Theory based counselling	Lay health advisor	Individualised care plan	Audit & Feedback	Psychology based counselling	Skill building	Financial incentive	Problem solving	Peer / Family support	Theory based counselling	Lay health advisor
**Articles examining teleeducation**																			
Arora, 2014					Y		Y		Y										
Avdal , 2011		Y	Y											Y		Y			
Bell, 2012			Y	Y		Y		Y											
Blackberry, 2013		Y	Y		Y		Y				Y	Y							
Bond, 2007		Y	Y	Y	Y		Y		Y		Y	Y							
Browning, 2016				Y															
Capozza, 2015		Y			Y				Y										
Davis, 2010		Y	Y		Y		Y				Y	Y							
Glasgow, 1997		Y	Y		Y		Y				Y	Y							
Glasgow, 2006		Y	Y	Y	Y		Y			Y	Y	Y							
Goodarzi, 2012					Y					Y		Y							
Graziano, 2009		Y	Y		Y		Y				Y	Y							
Islam, 2015					Y		Y		Y										
Kim, 2003		Y	Y								Y	Y							
Lee, 2007		Y	Y		Y						Y	Y							
Leu, 2005		Y																	
Liou, 2014					Y				Y		Y			Y					
Lorig, 2008					Y		Y				Y			Y					
McKay, 2002	Personalised coaching	Y	Y		Y									Y					
	Online peer support	Y	Y		Y		Y	Y		Y									
	Combination of both	Y	Y	Y	Y		Y	Y		Y									
Noh, 2010					Y						Y	Y							
Peimani, 2016	General SMS education			Y				Y			Y								
	Tailored SMS education	Y		Y				Y											

Piette, 2011		Y	Y	Y	Y				Y	Y									
Plotnikoff, 2013	Print materials with pedometer				Y														
	Print materials, pedometer & counselling			Y	Y				Y	Y									
Sacco, 2009		Y		Y	Y		Y		Y	Y	Y								
Shetty, 2011			Y		Y				Y		Y								
Whittemore, 2004		Y		Y	Y		Y		Y		Y	Y							
Williams, 2012		Y	Y		Y		Y		Y		Y	Y							
Wolever, 2010		Y	Y	Y	Y		Y		Y	Y	Y	Y							
Yoon, 2008		Y	Y		Y					Y	Y	Y							Y
Zolfaghari, 2011		Y	Y		Y				Y	Y	Y	Y		Y				Y	Y
**Articles examining telecase-management**																			
Anderson, 2011		Y	Y				Y		Y		Y	Y							
Aubert, 1998		Y	Y	Y	Y		Y		Y		Y	Y							
Glasgow, 2010	Computer assisted self-management (CASM)	Y			Y		Y				Y	Y							
	Enhanced CASM	Y	Y		Y		Y		Y										
Huizinga, 2010		Y	Y		Y		Y		Y		Y	Y							
Hussien, 2011		Y	Y		Y		Y	Y			Y	Y							
Piette, 2000		Y	Y		Y		Y		Y		Y	Y							
Ralston, 2009		Y	Y		Y		Y		Y		Y	Y							
Shea. 2009		Y	Y		Y		Y				Y	Y							
Wayne, 2015		Y	Y		Y		Y				Y	Y							
Zhou. 2014		Y	Y		Y		Y			Y	Y	Y							Y
**Articles examining telemonitoring**																			
Boaz, 2009		Y	Y				Y												
Bujnowska-Fedak, 2011		Y	Y									Y							
Cho, 2006		Y	Y						Y			Y							
Cho, 2009		Y	Y		Y						Y	Y							
Earle, 2010		Y	Y		Y									Y		Y			
Kim, 2016			Y																
Kwon, 2004		Y	Y		Y		Y				Y	Y							
Lee, 2015			Y		Y					Y		Y							
Lim, 2011	Self-monitoring only		Y								Y								
	U-healthcare group		Y		Y				Y										
Luley, 2011		Y	Y		Y					Y	Y	Y							
Nicolucci, 2015		Y	Y		Y						Y	Y							
Or, 2016		Y	Y																
Pressman, 2014		Y	Y		Y		Y				Y	Y							

Quinn, 2011	Monitoring with feedback		Y		Y														
	As above with unanalysed feedback from doctor	Y	Y		Y		Y				Y								
	As above with feedback on treatment	Y	Y		Y		Y												
Tildesley, 2011		Y	Y		Y		Y					Y							
Yoo , 2009		Y	Y		Y		Y					Y							
**Articles examining teleconsultation**																			
Ciemins, 2011		Y	Y		Y		Y				Y	Y							
Leichter, 2013		Y	Y		Y		Y				Y	Y							
Nagrebetsky, 2013		Y	Y		Y						Y	Y							
Rasmussen, 2015		Y	Y								Y	Y							
Smith, 2008		Y							Y					Y					
Shahid, 2015		Y	Y		Y						Y	Y							
Whitlock, 2000		Y					Y				Y								
Wong, 2005		Y	Y		Y		Y		Y		Y	Y							
**Articles examining telementoring**																			
Anderson-Loftin, 2005		Y			Y		Y	Y	Y		Y			Y					
Chan, 2014		Y	Y		Y		Y	Y			Y	Y		Y		Y			
Dale, 2009		Y	Y				Y				Y	Y							
Dang, 2013			Y		Y		Y	Y						Y					
Heisler, 2010			Y				Y	Y			Y	Y							
Keyserling, 2002	Clinic education with peer support	Y	Y	Y	Y		Y	Y			Y	Y							
	Clinic education	Y	Y	Y	Y		Y												
Long, 2012	Matched peer mentor support	Y	Y	Y				Y											
	Financial incentive					Y													
Samuel-Hodges, 2009		Y	Y	Y	Y		Y	Y	Y		Y								
Spenser, 2011		Y	Y		Y		Y	Y			Y								
Thom, 2013		Y	Y		Y		Y	Y			Y								
Walker, 2011		Y			Y		Y		Y					Y					
**Articles examining teleeducation & telecase-management**																			
Carter, 2011		Y	Y	Y	Y		Y				Y	Y							
Cho, 2011		Y	Y				Y				Y	Y							
Crowley, 2013		Y	Y	Y	Y		Y		Y		Y								
Holmen, 2014	Education only		Y								Y	Y							

	Education with case-management	Y	Y	Y			Y												
Maljanian, 2005		Y			Y				Y		Y	Y							
Nesari, 2010		Y	Y		Y		Y		Y		Y	Y							
Oh , 2003		Y	Y		Y		Y				Y	Y							
Piette, 2001		Y	Y		Y		Y				Y	Y							
Schillinger, 2009	Automated telephone self-management support	Y	Y	Y	Y				Y		Y								
	Group medical visits to physician and health educators facilitation	Y		Y	Y	Y			Y										
Tjam, 2006		Y	Y		Y		Y		Y		Y	Y							
Young, 2005		Y	Y	Y	Y		Y		Y	Y	Y	Y							
**Articles examining teleeducation & telemonitoring**																			
Chen, 2011		Y	Y		Y		Y		Y					Y		Y		Y	
Faridi, 2008		Y	Y		Y							Y							
Greenwood, 2015		Y	Y		Y		Y		Y		Y	Y		Y					
Karhula, 2015		Y	Y		Y		Y				Y	Y							
Lim, 2016			Y																
Steventon, 2015		Y	Y		Y					Y	Y								
Tang, 2013		Y	Y		Y		Y		Y		Y	Y							
Wakefield, 2011	High intensity	Y	Y		Y		Y		Y	Y	Y								
	Low intensity	Y	Y		Y		Y		Y	Y									
**Articles examining telecase-management & telemonitoring**																			
Anzaldo-Campos, 2016	Peer support and care management	Y						Y		Y									
	Peer support, care management & monitoring	Y	Y					Y		Y									
Castelnuovo, 2011		Y	Y	Y	Y		Y		Y		Y								
Del Prato, 2012		y	Y				Y			Y	Y	Y							
Kim, 2008		Y	Y		Y						Y	Y							
Kim, 2009		Y	Y		Y		Y				Y	Y							
McMahon, 2005		y	Y		Y						Y	Y							
McMahon, 2012	Online care group	Y	Y		Y		Y				Y	Y		Y			Y		
	Telephone care group	Y	Y		Y		Y				Y	Y		Y			Y		
Orsama, 2013		y	Y		Y		Y		Y		Y	Y							
Rodriguez-Idigoras, 2009		y	Y				Y				Y	Y							
Stone, 2010		y	Y		Y						Y	Y							

Stone, 2012	Care coordination calls	Y	Y		Y						Y	Y							
	Active care with home monitoring	Y	Y		Y														
Waki, 2014		Y	Y								Y	Y							
**Articles examining telecase-management & telemonitoring**																			
Hsu, 2016		Y	Y		Y		Y				Y								

Table S5: Details of educational content delivered to participants in studies examining teleeducation

Author		Intervention						Control					
		Diabetes management	Diet	Diabetes complications (foot care, retinopathy)	Diabetes symptoms (e.g. hypoglycaemia)	Medication adherence	Education trivia / challenge	Diabetes management	Diet	Diabetes complications (foot care, retinopathy)	Diabetes symptoms (e.g. hypoglycaemia)	Medication adherence	Education trivia / challenge
**Articles examining teleeducation**													
Arora, 2014		Y	Y	Y	Y	Y	Y						
Avdal , 2011													
Bell, 2012		Y	Y										
Blackberry, 2013		Y											
Bond, 2007		Y	Y					Y	Y				
Browning, 2016		Y											
Capozza, 2015		Y	Y		Y	Y							
Davis, 2010		Y	Y										
Glasgow, 1997			Y										
Glasgow, 2006			Y										
Goodarzi, 2012		Y	Y			Y							
Graziano, 2009		Y	Y			Y		Y	Y				
Islam, 2015		Y	Y	Y		Y							
Kim, 2003		Y	Y		Y	Y							
Lee, 2007		Y	Y	Y	Y	y							
Leu, 2005			Y			Y							
Liou, 2014		Y	Y	Y	Y								
Lorig, 2008		Y						Y					
McKay, 2002	Personalised coaching		Y						Y				
	Online peer support		Y						Y				
	Combination of both	Y	Y					Y	Y				
Noh, 2010		Y	Y		Y								
Peimani, 2016	General SMS education	Y	Y			Y							
	Tailored SMS education	Y	Y			Y							
Piette, 2011		Y											
Plotnikoff, 2013	Print materials with pedometer	Y											

	Print materials, pedometer & counselling	Y	Y	Y	Y	Y							
Sacco, 2009		Y	Y	Y		Y							
Shetty, 2011		Y	Y			Y		Y	Y			Y	
Whittemore, 2004		Y											
Williams, 2012		Y	Y			Y							
Wolever, 2010		Y	Y			Y							
Yoon, 2008		Y											
Zolfaghari, 2011		Y	Y	Y	Y	Y		Y	Y			Y	
**Articles examining teleeducation & telemonitoring**													
Chen, 2011		Y			Y			Y			Y		
Faridi, 2008		Y						Y					
Greenwood, 2015		Y	Y			Y							
Karhula, 2015		Y											
Lim, 2016		Y	Y					Y	Y				
Steventon, 2015		Y											
Tang, 2013		Y	Y			Y							
Wakefield, 2011	High intensity	Y	Y	Y		Y							
	Low intensity	Y	Y	Y		Y							
**Articles examining teleeducation & telecase-management**													
Carter, 2011		Y	Y										
Cho, 2011		Y	Y										
Crowley, 2013		Y	Y		Y								
Holmen, 2014	Education only	Y											
	Education with case-management	Y	Y										
Maljanian, 2005		Y	Y	Y	Y			Y	Y				
Nesari, 2010		Y	Y	Y	Y	Y		Y	Y	Y	Y		
Oh , 2003		Y	Y										
Piette, 2001		Y				Y		Y					
Schillinger, 2009	Automated telephone self-management support	Y	Y		Y								
	Group medical visits to physician and health educators facilitation												
Tjam, 2006				Y	Y	Y		Y	Y		Y		
Young, 2005		Y	Y			Y							

Table S6: Details of self-monitoring frequency and recommended schedule

Author	Intervention				Control			
	Monitoring frequency (Target readings per week)	SMBG Schedule	No of days over which monitoring is spread	Person delivering meter training	Monitoring frequency (Target readings per week)	SMBG Schedule	No of days over which monitoring is spread	Person delivering meter training
**Articles examining telemonitoring**								
Boaz, 2009	Monitor six times daily for at least 2x/week and more than 3x/day for the rest							
Bujnowska-Fedak, 2011				Nurse				
Cho, 2006	Depending on glucose control							
Cho, 2009	Three times a day for insulin treated and twice daily for OHA				Three times a day for insulin treated and twice daily for OHA			
Earle, 2010	Four to nine times							
Kim, 2016	Three times daily to twice weekly depending on medication and HbA1c	Min twice weekly if HbA1c<7% & not on medications, daily for those with HbA1c<7% but	1 week					

			on medications, more than once daily if HbA1c>7%, twice daily if HbA1c<7% & on insulin and min 3 times daily if HbA1c>7% & on insulin						
Kwon, 2004			Fasting and post-prandial						
Lee, 2015		Five times per day	Sahur, post-2–h Sahur, midday, before breaking of fast and Iftar	Daily		Five times per day	Sahur, post-2–h Sahur, midday, before breaking of fast and Iftar	Daily	
Lim, 2011	Self-monitoring only	Eight times	≥3 at fasting, ≥3 postprandial, and ≥2 bed-times		Trainers	Eight times	≥3 at fasting, ≥3 postprandial, and ≥2 bed-times		
	U-healthcare group								
Luley, 2011		Daily							
Nicolucci, 2015					Nurse				
Or, 2016									
Pressman, 2014									
Quinn, 2011	Home monitoring with feedback	System driven based upon blood glucose results					As recommended by physician		
	As above with unanalysed feedback from doctor								
	As above with feedback on treatment								
Tildesley, 2011		At least 3 times daily				At least 3 times daily			
Yoo , 2009		Twice daily	Before breakfast and bedtime	Daily					
**Articles examining telecase-management & telemonitoring**									

Anzaldo-Campos, 2016	Peer support and care management								
	Peer support, care management & monitoring	Twice daily for 1st month, then twice weekly thereafter	Fasting and post prandial	Daily for first month and two days in a week thereafter	Nurse				
Del Prato, 2012		Six target readings	Pre and 2h post breakfast , lunch and supper	2 days		Six target readings	Pre and 2h post breakfast , lunch and supper	2 days	
Kim, 2008		Minimum once weekly	Breakfast, lunch and dinner						
Kim, 2009									
McMahon, 2005		Min 3 times							
McMahon, 2012	Online care group	Individualized according to patient glucose levels							
	Telephone care group								
Orsama, 2013		Three days each week	Pre and post prandial						
Rodriguez-Idigoras, 2009									
Stone, 2010		At least once daily		Daily					
Stone, 2012	Care coordination calls								
	Active care with home monitoring	At least once daily		Daily					
Waki, 2014		Twice daily							
**Articles examining teleeducation & telemonitoring**									
Chen, 2011		Once or twice daily	Fasting plasma glucose, pre-lunch or pre dinner and 2 h post prandial						
Faridi, 2008		Once daily	Upon awakening	Daily					
Greenwood, 2015			Before and 2 hours post meal			None specified			
Karhula, 2015		At least once weekly							
Lim, 2016		At least 8 times a week	Min 3 fasting, 3 post-prandial, 2 at bedtime	1 week		At least 8 times a week	Min 3 fasting, 3 post-prandial, 2 at bedtime	1 week	
Steventon, 2015		Up to 5 days per week	Same time each day	Five		As agreed with physician			
Tang, 2013									

Wakefield, 2011	High intensity	As directed by physician							
	Low intensity	As directed by physician							

SMBG- Self-monitoring of blood glucose

Table S7: Details of the action(s) taken based upon results from glucose monitoring

Author	Intervention				Control			
	Action taken according to SMBG results	Parameter / Trigger for action	Interventionist	Area of changes made	Action taken according to SMBG results	Parameter / Trigger for action	Interventionist	Area of changes made
**Articles examining telemonitoring**								
Boaz, 2009	Text message and call to patient on medical advice	50 to 280 mg/dL	Diabetes nurse	Titration of insulin dose, oral medication				
Bujnowska-Fedak, 2011	Call to patient on medical advice	50 to 280 mg/dL	General practitioner	Titration of insulin dose, oral medications, diet,				
Cho, 2006	Appropriate recommendations based upon glucose data every 2 weeks			As required including referral to nurse or dietician for issues related to self-management or lifestyle changes				
Cho, 2009	Recommendation by Internet				Recommendation by SMS			

Earle, 2010	Recommendations to alter treatment and letter to GP			Alter treatment				
Kim, 2016	Recommendations on blood glucose control or telephone calls							
Kwon, 2004	Recommendation based upon history, smoking habits, laboratory data and glucose levels		Endocrinologist, nurses, dieticians	Medication dosage change, Lifestyle modification and medical nutrition therapy	Recommendation during the 2-3 clinic visits which could include dietary advice and lifestyle modification	As determined by clinician during visit	Endocrinologist	Lifestyle modifications
Lee, 2015	Feedback on blood results and goal setting	≤ 3.9 mmol/l and ≥ 11.1 mmol/l.		Goal setting				
Lim, 2011	Automated messages which were patients specific based upon glucose results	<4mmol/L or >8mmol/L		Changes to medications, lifestyle advice,				
Luley, 2011	Weekly reports sent to patients to motivate them							
Nicolucci, 2015	Monthly calls to patient	When patients forget to enter or predefined alarm values are recorded	Nurse	Self-monitoring results, barriers to compliance, causes of inadequate metabolic or blood pressure control				
Or, 2016	Abnormal readings display							
Pressman, 2014	Weekly messages to patients		Care-manager	Educational message				
Quinn, 2011 — Monitoring with feedback	Electronic action plan every 2.5 month prior to clinic visit		Diabetes educators	Educational, behavioural, and motivational message	Electronic action plan every 2.5 month prior to clinic visit			
Quinn, 2011 — As above with unanalysed feedback from doctor								
Quinn, 2011 — As above with feedback on treatment								
Tildesley, 2011				Change in insulin dosage, testing frequency,				

					motivational message				
Yoo , 2009 **Articles examining telecase management & telemonitoring**		Immediate feedback message			Variable, but include motivational, reminders or educational message				
**Articles examining telecase management & telemonitoring**									
Anzaldo-Campos, 2016	Peer support and care management								
	Peer support, care management & monitoring	Identification of low or high readings							
Del Prato, 2012		Dosage adjustment of metformin up to 2g/day	>5.6mmol/L		Medications				
Kim, 2008		Feedback based upon reported data including dosage adjustment and motivational messages		Diabetic educator, Nursing college academician	Medication, exercise, education and self-monitoring				
Kim, 2009		Monthly measurement reports generated to facilitate communication between participant and nurse			Problem solving on patient issues and emotional support				

McMahon, 2005		Respond to any queries by patients and communicated to primary care provider on results		Diabetes educator	Glucose treatment algorithm				
McMahon, 2012	Online care group	Reinforcement of modification advice and medication change		Nurse, Pharmacist	Medication, Lifestyle and nutritional advice				
	Telephone care group								
Orsama, 2013		Specific algorithm and feedback based upon reported data			Motivational, behavioural skills to support patient care				
Rodriguez-Idigoras, 2009		Alarm to call centre		Physician, Diabetes nurse	Standard protocol (not specified)				
Stone, 2010		Individualized telephone call		Nurse practitioner	Self-management education				
Stone, 2012	Care coordination calls								
	Active care with home monitoring	Individualized telephone call	<50mg/dL or >300mg/dL for 72hr	Nurse practitioner	Self-management education				
Waki, 2014		Alerts for missed, late readings or readings outside threshold	<40mg/dL or >400mg/dL		Motivational				
**Articles examining teleeducation & telemonitoring**									
Chen, 2011				Diabetes educator	Education and insulin dosage adjustment				
Faridi, 2008					Tailored feedback and reminders				
Greenwood, 2015		Feedback on glucose results	<50mg/dL or >450mg/dL		SMBG trend, patterns and goals, medication option and changes				
Karhula, 2015					Knowledge on chronic disease				
Lim, 2016		Tailored message depending on glucose levels			Dietary, exercise				

Steventon, 2015		Feedback from provider		Nurse, Matron	Home visit, medication titration, advice on disease management and referral to doctor				
Tang, 2013		Regular feedback on clinical variable			Personalized educational message				
Wakefield, 2011	High intensity	Summary data generated for review by interventionist		Nurse	Additional health information, increased monitoring, compliance strategies, problem resolution facilitation or contact with subject physician				
	Low intensity								

SMBG- Self-monitoring of blood glucose

Table S8: Results of subgroup and sensitivity analysis for primary outcome

Subgroup	No of studies	Sample size	Fixed effects (95% CI)	Random effects (95% CI)
**Telemedicine**	93	16,791	-0.61 (-0.61 to -0.60)	-0.43 (-0.64 to -0.21)
**Study location**				
Asia	31	4,316	-0.85 (-0.86 to -0.84)	-0.85 (-1.34 to -0.36)
Europe	14	2,176	-0.05 (-0.07 to -0.04)	-0.18 (-0.36 to 0.00)
North America	47	10,199	-0.17 (-0.19 to -0.16)	-0.23 (-0.37 to -0.10)
Australia	1	120	0.60 (0.22 to 0.98)	0.60 (0.22 to 0.98)
**Study sample size**				
<100 participants	39	2,292	-0.26 (-0.30 to -0.23)	-0.52 (-0.67 to -0.36)
101-200 participants	22	2,913	-0.26 (-0.32 to -0.20)	-0.40 (-0.74 to -0.05)
>200 participants	32	11,606	-0.62 (-0.62 to -0.61)	-0.31 (-0.67 to 0.05)
**Study duration**				
≤3 months	17	1,377	-0.54 (-0.67 to -0.41)	-0.67 (-0.93 to -0.41)
4-6 months	36	4,538	-1.26 (-1.27 to -1.25)	-0.41 (-0.84 to 0.02)
7-11 months	4	659	-0.54 (-0.79 to -0.29)	-0.66 (-1.18 to -0.15)
≥ 12 months	36	10,237	-0.06 (-0.07 to -0.05)	-0.26 (-0.40 to -0.12)
**Study participants baseline HbA1c**				
<8.0%	48	5,720	-0.22 (-0.25 to -0.19)	-0.45 (-0.59 to -0.31)
≥8.0%	45	8,100	-0.60 (-0.61 to -0.60)	-0.36 (-0.73 to -0.03)
**Medium of communication used**				
Telephone	35	7,323	-0.06 (-0.07 to -0.06)	-0.29 (-0.42 to -0.16)
Computer	3	648	0.04 (-0.20 to 0.27)	0.04 (-0.20 to 0.27)
Mobile phone	7	781	-1.93 (-1.94 to -1.92)	-0.47 (-1.53 to 0.58)
Handheld device	10	1,479	-0.05 (-0.07 to -0.04)	-0.29 (-0.50 to -0.08)
SMS	9	1,012	-0.59 (-0.77 to -0.40)	-0.65 (-1.05 to -0.25)
Web-based intervention	17	2,528	-0.23 (-0.27 to -0.19)	-0.49 (-0.66 to -0.31)
Telephone & mobile device	4	594	-0.25 (-0.47 to -0.03)	-0.39 (-0.96 to 0.18)
Web-based & handheld device	4	1,952	-0.34 (-0.43 to -0.24)	-0.45 (-0.68 to -0.21)
Web-based & telephone	3	428	-0.23 (-0.42 to -0.04)	-0.37 (-0.88 to 0.13)
**Year study conducted**				
Before 2000	3	276	-1.07 (-1.20 to -0.95)	-1.01 (-1.26 to -0.75)
2000-2004	6	777	0.42 (0.31 to 0.53)	-0.48 (-1.34 to 0.39)
2005-2009	25	5,054	-0.12 (-0.16 to -0.09)	-0.24 (-0.37 to -0.11)
2010 onwards	58	10,664	-0.62 (-0.63 to -0.62)	-0.42 (-0.70 to -0.15)
**Telemedicine strategy**				
Telemonitoring	14	1,577	-0.49 (-0.60 to -0.39)	-0.44 (-0.66 to -0.22)
Teleconsultation	7	1,328	-1.94 (-1.95 to -1.93)	-0.64 (-1.71 to 0.43)
Tele-education	26	4,211	-0.28 (-0.37 to -0.19)	-0.36 (-0.58 to -0.15)
Telecase-management	8	2,620	-0.44 (-0.52 to -0.36)	-0.28 (-0.78 to 0.22)
Telementoring	11	2,892	-0.08 (-0.09 to -0.08)	-0.28 (-0.52 to -0.04)
Tele-education & telemonitoring	8	1,540	-0.32 (-0.42 to -0.22)	-0.35 (-0.56 to -0.14)
Telecase-management & telemonitoring	9	1,194	-0.05 (-0.07 to -0.04)	-0.54 (-0.85 to -0.23)

Tele-education & telecase-management	9	1,409	0.04 (0.02 to 0.06)	-0.31 (-0.51 to -0.10)
**Independent medication change**				
Allowed	30	5,443	-0.13 (-0.15 to -0.12)	-0.35 (-0.51 to -0.20)
Not allowed / Not reported	62	11,428	-0.69 (-0.69 to -0.68)	-0.43 (-0.71 to -0.14)

Table S9 – Results of the network meta-analyses

All estimates are presented as weighted mean difference (WMD) with the corresponding 95% confidence intervals. Comparisons between treatments should be read from column to row and the estimate is in the cell in common between the column-defining treatment and the row-defining treatment. Weighted mean difference that are negative favor the column-defining treatment. To obtain odds ratios for comparisons in the opposite direction, reciprocals should be taken. All significant results are presented in bold and underlined.

Table S9a: Pairwise meta-analyses and network meta-analyses of the various telemedicine strategies on glycosylated haemoglobin. Results of effectiveness of intervention are presented as mean difference (95% confidence interval) for each pairwise comparison of intervention, based on direct evidence alone (lower-left triangle) or direct and indirect evidence (upper right triangle)

Usual care	**-0.37 (-0.59,-0.15)**	-0.24 (-0.61,0.14)	**-0.71 (-1.23,-0.20)**	**-0.40 (-0.70,-0.11)**	-0.28 (-0.61,0.04)	**-0.41 (-0.79,-0.02)**	**-0.58 (-0.96,-0.19)**	**-0.43 (-0.80,-0.06)**	-1.20 (-2.70,0.30)
**-0.36 (-0.58, -0.15)**	Teleeducation	0.13 (-0.29,0.55)	-0.35 (-0.91,0.21)	-0.03 (-0.40,0.34)	0.09 (-0.31,0.48)	-0.04 (-0.48,0.41)	-0.21 (-0.65,0.24)	-0.06 (-0.49,0.36)	-0.83 (-2.35,0.69)
-0.28 (-0.78, 0.22)	-0.40 (-1.03, 0.23)	Telecase-management	-0.48 (-1.11,0.16)	-0.16 (-0.64,0.31)	-0.04 (-0.54,0.45)	-0.17 (-0.71,0.37)	-0.34 (-0.87,0.20)	-0.19 (-0.72,0.33)	-0.96 (-2.51,0.59)
-0.64 (-1.71, 0.43)	ND	ND	Teleconsultation	0.31 (-0.28,0.91)	0.43 (-0.18,1.04)	0.31 (-0.34,0.95)	0.14 (-0.50,0.78)	0.28 (-0.35,0.92)	-0.49 (-2.07,1.10)
**-0.44 (-0.66, -0.22)**	ND	ND	ND	Telemonitoring	0.12 (-0.32,0.56)	-0.01 (-0.48,0.47)	-0.17 (-0.66,0.31)	-0.03 (-0.50,0.44)	-0.80 (-2.33,0.73)
**-0.28 (-0.52, -0.04)**	ND	ND	ND	ND	Telementoring	-0.12 (-0.63,0.38)	-0.29 (-0.80,0.21)	-0.15 (-0.64,0.34)	-0.92 (-2.45,0.62)
**-0.35 (-0.56, -0.14)**	ND	ND	ND	0.02 (-0.37, 0.41)	ND	Teleeducation & telemonitoring	-0.17 (-0.71,0.38)	-0.02 (-0.56,0.51)	-0.79 (-2.34,0.76)
**-0.54 (-0.85, -0.23)**	-0.16 (-0.70, 0.38)	ND	ND	ND	ND	ND	Telecase-management & Telemonitoring	0.14 (-0.39,0.68)	-0.62 (-2.18,0.93)
**-0.31 (-0.51, -0.10)**	ND	ND	ND	ND	ND	ND	ND	Teleeducation & Telecase-management	-0.77 (-2.32,0.78)
**-1.20 (-2.30, -0.10)**	ND	ND	ND	ND	ND	ND	ND	ND	Telecase-management & Teleconsultation

ND- No data

Lower left results compare row-defining intervention against column-defining intervention. Upper right results compare column-defining intervention against row-defining interventions.

Table S9b: Network estimated weighted mean difference (95% confidence interval) of different telemedicine strategies on fasting plasma glucose

Usual care	**-0.71 (-1.35,-0.07)**	**-1.77 (-2.76,-0.77)**	**-0.82 (-1.46,-0.19)**	**-1.24 (-2.18,-0.30)**	-0.26 (-1.69,1.16)	-0.70 (-2.27,0.87)
	Teleeducation	-1.06 (-2.24,0.12)	-0.11 (-1.02,0.79)	-0.53 (-1.67,0.60)	0.45 (-1.11,2.01)	0.01 (-1.69,1.71)
		Telecase-management	0.95 (-0.24,2.13)	0.53 (-0.84,1.90)	1.51 (-0.23,3.24)	1.07 (-0.79,2.93)
			Telemonitoring	-0.42 (-1.56,0.72)	0.56 (-1.00,2.12)	0.12 (-1.57,1.82)
				Teleeducation & Telemonitoring	0.98 (-0.73,2.69)	0.54 (-1.29,2.37)
					Telecase-management & Telemonitoring	-0.44 (-2.56,1.68)
						Teleeducation & Telecase-management

Table S9c: Network estimated weighted mean difference (95% confidence interval) of different telemedicine strategies on systolic blood pressure

Usual care	**-3.74 (-5.89,-1.59)**	-2.73 (-5.55,0.09)	-0.28 (-2.73,2.17)	-0.41 (-3.02,2.19)	0.93 (-0.93,2.78)	-2.65 (-5.95,0.65)	-1.98 (-5.54,1.58)	1.09 (-2.32,4.51)
	Teleeducation	1.01 (-2.38,4.40)	**3.46 (0.21,6.71)**	3.33 (-0.06,6.72)	**4.67 (1.84,7.50)**	1.09 (-2.89,5.07)	1.76 (-2.39,5.91)	**4.84 (0.74,8.93)**
		Telecase-management	2.45 (-1.29,6.18)	2.32 (-1.53,6.16)	**3.66 (0.28,7.03)**	0.08 (-4.28,4.43)	0.75 (-3.79,5.29)	3.83 (-0.63,8.28)
			Teleconsultation	-0.13 (-3.72,3.46)	1.21 (-1.85,4.27)	-2.37 (-6.53,1.79)	-1.70 (-6.01,2.61)	1.38 (-2.89,5.65)
				Telemonitoring	1.34 (-1.87,4.55)	-2.24 (-6.37,1.88)	-1.57 (-5.99,2.85)	1.51 (-2.67,5.69)
					Telementoring	-3.58 (-7.39,0.23)	-2.91 (-6.92,1.10)	0.17 (-3.76,4.09)
						Teleeducation & telemonitoring	0.67 (-4.21,5.56)	3.75 (-0.73,8.22)
							Telecase-management & Telemonitoring	3.08 (-1.90,8.06)
								Teleeducation & Telecase-management

Table S9d: Network estimated weighted mean difference (95% confidence interval) of different telemedicine strategies on diastolic blood pressure

Usual care	**-1.67 (-3.08,-0.26)**	-1.65 (-3.37,0.06)	**-4.77 (-7.02,-2.51)**	-0.90 (-2.80,1.00)	1.16 (-0.16,2.47)	-0.90 (-3.32,1.53)	-1.66 (-3.87,0.55)	3.00 (-0.92,6.92)
	Teleeducation	0.01 (-2.12,2.14)	**-3.10 (-5.82,-0.37)**	0.76 (-1.57,3.09)	**2.83 (0.91,4.74)**	0.77 (-2.02,3.56)	0.01 (-2.62,2.63)	**4.67 (0.50,8.83)**
		Telecase-management	**-3.11 (-5.98,-0.24)**	0.75 (-1.79,3.29)	**2.81 (0.66,4.97)**	0.76 (-2.21,3.72)	-0.01 (-2.81,2.80)	**4.65 (0.38,8.93)**
			Teleconsultation	**3.86 (0.79,6.94)**	**5.92 (3.26,8.59)**	**3.87 (0.50,7.24)**	3.10 (-0.04,6.25)	**7.77 (3.24,12.29)**
				Telemonitoring	2.06 (-0.22,4.34)	0.01 (-3.04,3.05)	-0.76 (-3.68,2.17)	3.90 (-0.45,8.26)
					Telementoring	-2.06 (-4.80,0.69)	**-2.82 (-5.40,-0.24)**	1.84 (-2.29,5.97)
						Teleeducation & telemonitoring	-0.76 (-4.05,2.52)	3.90 (-0.71,8.51)
							Telecase-management & Telemonitoring	**4.66 (0.16,9.16)**
								Teleeducation & Telecase-management

Table S9e: Network estimated weighted mean difference (95% confidence interval) of different telemedicine strategies on body mass index

Usual care	-0.22 (-1.11,0.67)	0.60 (-0.34,1.54)	0.35 (-0.90,1.61)	**-0.97 (-1.80,-0.15)**	-0.40 (-1.34,0.53)	-1.49 (-3.42,0.45)	0.11 (-1.06,1.29)	0.09 (-1.69,1.86)
	Teleeducation	0.82 (-0.41,2.05)	0.57 (-0.97,2.11)	-0.75 (-1.96,0.46)	-0.18 (-1.47,1.11)	-1.27 (-3.39,0.86)	0.33 (-1.14,1.81)	0.31 (-1.68,2.29)
		Telecase-management	-0.25 (-1.82,1.32)	**-1.57 (-2.82,-0.32)**	-1.00 (-2.33,0.32)	-2.08 (-4.23,0.06)	-0.48 (-1.99,1.02)	-0.51 (-2.52,1.50)
			Teleconsultation	-1.32 (-2.83,0.18)	-0.75 (-2.32,0.81)	-1.84 (-4.14,0.47)	-0.24 (-1.96,1.49)	-0.26 (-2.44,1.91)
				Telemonitoring	0.57 (-0.68,1.82)	-0.51 (-2.61,1.59)	1.09 (-0.35,2.53)	1.06 (-0.90,3.02)
					Telementoring	-1.08 (-3.23,1.06)	0.52 (-0.99,2.02)	0.49 (-1.52,2.50)
						Teleeducation & telemonitoring	1.60 (-0.66,3.86)	1.57 (-1.05,4.20)
							Telecase-management & Telemonitoring	-0.03 (-2.16,2.10)
								Teleeducation & Telecase-management

Table S9f: Network estimated weighted mean difference (95% confidence interval) of different telemedicine strategies on total cholesterol

Usual care	-0.15 (-0.42,0.12)	-0.03 (-0.50,0.44)	0.00 (-0.31,0.32)	0.01 (-0.50,0.53)	-0.27 (-1.18,0.64)	-0.25 (-0.90,0.40)	0.12 (-0.58,0.83)
	Teleeducation	0.12 (-0.38,0.63)	0.15 (-0.26,0.57)	0.16 (-0.42,0.74)	-0.12 (-1.07,0.83)	-0.10 (-0.80,0.61)	0.27 (-0.48,1.03)
		Telecase-management	0.03 (-0.54,0.59)	0.04 (-0.66,0.73)	-0.24 (-1.27,0.78)	-0.22 (-1.02,0.58)	0.15 (-0.70,0.99)
			Telemonitoring	0.01 (-0.59,0.61)	-0.27 (-1.24,0.69)	-0.25 (-0.97,0.47)	0.12 (-0.65,0.89)
				Telementoring	-0.28 (-1.33,0.76)	-0.26 (-1.09,0.57)	0.11 (-0.76,0.98)
					Teleeducation & telemonitoring	0.02 (-1.10,1.14)	0.39 (-0.76,1.54)
						Telecase-management & Telemonitoring	0.37 (-0.59,1.33)
							Teleeducation & Telecase-management

Table S9g: Network estimated weighted mean difference (95% confidence interval) of different telemedicine strategies on low-density lipoprotein

Usual care	0.00 (-0.13,0.13)	0.03 (-0.14,0.20)	-0.20 (-0.40,0.00)	-0.06 (-0.20,0.07)	-0.06 (-0.24,0.13)	-0.06 (-0.30,0.17)	0.03 (-0.26,0.32)
	Teleeducation	0.03 (-0.17,0.22)	-0.20 (-0.44,0.03)	-0.07 (-0.26,0.12)	-0.06 (-0.28,0.16)	-0.07 (-0.33,0.20)	0.03 (-0.29,0.34)
		Telecase-management	-0.23 (-0.49,0.03)	-0.09 (-0.31,0.12)	-0.09 (-0.33,0.16)	-0.09 (-0.38,0.19)	0.00 (-0.33,0.33)
			Teleconsultation	0.14 (-0.10,0.37)	0.14 (-0.12,0.41)	0.14 (-0.17,0.44)	0.23 (-0.12,0.58)
				Telemonitoring	0.01 (-0.22,0.23)	0.00 (-0.27,0.27)	0.09 (-0.22,0.41)
					Telementoring	-0.01 (-0.30,0.29)	0.09 (-0.25,0.43)
						Telecase-management & Telemonitoring	0.09 (-0.28,0.46)
							Teleeducation & Telecase-management

Table S9h: Network estimated weighted mean difference (95% confidence interval) of different telemedicine strategies on high-density lipoprotein

Usual care	0.01 (-0.04,0.06)	0.01 (-0.08,0.09)	**0.70 (0.51,0.88)**	0.00 (-0.05,0.06)	-0.06 (-0.14,0.03)	**-0.13 (-0.23,-0.03)**	0.10 (-0.11,0.31)
	Teleeducation	0.00 (-0.09,0.09)	**0.69 (0.50,0.89)**	0.00 (-0.08,0.07)	-0.07 (-0.16,0.03)	**-0.14 (-0.25,-0.03)**	0.09 (-0.12,0.31)
		Telecase-management	**0.69 (0.49,0.89)**	0.00 (-0.10,0.10)	-0.07 (-0.19,0.05)	**-0.14 (-0.27,-0.01)**	0.09 (-0.13,0.32)
			Teleconsultation	**-0.69 (-0.89,-0.50)**	**-0.76 (-0.96,-0.55)**	**-0.83 (-1.04,-0.62)**	**-0.60 (-0.88,-0.32)**
				Telemonitoring	-0.06 (-0.17,0.04)	**-0.14 (-0.25,-0.02)**	0.10 (-0.12,0.31)
					Telementoring	-0.07 (-0.20,0.06)	0.16 (-0.06,0.38)
						Telecase-management & Telemonitoring	0.23 (0.00,0.46)
							Teleeducation & Telecase-management

Table S9i: Network estimated weighted mean difference (95% confidence interval) of different telemedicine strategies on triglycerides

Usual care	**-0.14 (-0.22,-0.06)**	0.01 (-0.14,0.17)	-0.09 (-0.37,0.20)	-0.02 (-0.15,0.12)	**0.20 (0.07,0.33)**	-0.17 (-0.56,0.22)	-0.24 (-0.51,0.03)	-0.07 (-0.55,0.40)
	Teleeducation	0.15 (-0.02,0.33)	0.06 (-0.24,0.35)	0.13 (-0.03,0.28)	**0.34 (0.19,0.49)**	-0.03 (-0.42,0.37)	-0.10 (-0.38,0.18)	0.07 (-0.41,0.55)
		Telecase-management	-0.10 (-0.42,0.23)	-0.03 (-0.23,0.17)	0.19 (-0.02,0.39)	-0.18 (-0.60,0.24)	-0.25 (-0.56,0.06)	-0.08 (-0.58,0.41)
			Teleconsultation	0.07 (-0.25,0.38)	0.29 (-0.03,0.60)	-0.08 (-0.57,0.40)	-0.16 (-0.55,0.24)	0.01 (-0.54,0.57)
				Telemonitoring	**0.22 (0.03,0.40)**	-0.15 (-0.56,0.26)	-0.23 (-0.52,0.07)	-0.06 (-0.55,0.44)
					Telementoring	-0.37 (-0.78,0.04)	**-0.44 (-0.74,-0.14)**	-0.27 (-0.76,0.22)
						Teleeducation & Telemonitoring	-0.07 (-0.54,0.40)	0.10 (-0.51,0.71)
							Telecase-management & Telemonitoring	0.17 (-0.37,0.71)
								Teleeducation & Telecase-management

Table S10: Inconsistency estimates

In all network meta-analysis, transitivity is the crucial assumption since it permits the use of indirect evidences, i.e. permits the comparison of treatment that never been directly contrasted. To estimate the inconsistency or transitivity, we used the difference between direct and indirect estimates (called inconsistency factor IF) and the corresponding 95% CI for each IF in each closed triangular loop. An inconsistent loop is those that present inconsistency factors with 95% confidence intervals incompatible with zero. For example, for the triangular loop of evidence including usual care-teleeducation-telecase-management for total cholesterol, the weighted mean difference for estimated effect differs between direct and indirect evidence by 0.03, however the 95% confidence interval includes zero indicating the possibility of no difference.

To further supplement our analyses on transitivity, we used the design-by-treatment interaction model which provides a single inference, using the χ^2 test, about the plausibility of assuming consistency throughout the entire network

Table S10a: Inconsistency of treatment triangular loop for each outcome network

Closed triangular loop of evidence	Inconsistency factor (95% confidence interval)	Loop heterogeneity tau^2
**Glycosylated haemoglobin**		
Usual care – Teleeducation – Telecase-management	0.32 (0.00, 1.71)	0.35
Usual care – Teleeducation – Teleeducation & Telecase-management	0.17 (0.00, 1.43)	0.04
Usual care – Telemonitoring – Teleeducation & Telemonitoring	0.11 (0.00, 0.91)	0.08
**Total cholesterol**		
Usual care – Teleeducation – Telecase-management	0.03 (0.00, 0.70)	0.03
**Low-density lipoprotein**		
Usual care – Teleeducation – Telecase-management	0.16 (0.00, 0.67)	0.03
**High-density lipoprotein**		
Usual care – Teleeducation – Telecase-management	0.05 (0.00, 0.21)	0.001
**Triglyceride**		
Usual care – Teleeducation – Telecase-management	0.20 (0.00, 0.73)	0.004
**Body mass index**		
Usual care – Teleeducation – Telecase-management	0.84 (0.00, 3.63)	0.18
**Systolic blood pressure**		
Usual care – Teleeducation – Telecase-management	4.11 (0.00, 11.87)	<0.001
**Diastolic blood pressure**		
Usual care – Teleeducation – Telecase-management	3.83 (0.00, 10.26)	1.31

Table S10b: Assessment of global inconsistency in networks using the 'design-by-treatment' interaction model

Network outcome	Chi-square	P-value for test of global inconsistency
Glycosylated haemoglobin	2.71	0.74
Total cholesterol	0.01	0.91
Low-density lipoprotein	0.39	0.53
High-density lipoprotein	0.18	0.67
Triglyceride	0.53	0.47
Body mass index	0.34	0.56
Systolic blood pressure	0.95	0.33
Diastolic blood pressure	1.58	0.21

Table S11: Summary of effect sizes comparing meta-analysis from other studies and current study

Reference list of studies used in the current comparison

1. Flodgren G, Rachas A, Farmer AJ, et al. Interactive telemedicine: effects on professional practice and health care outcomes. Cochrane Database of Systematic Reviews 2015(9). Art. No.: CD002098. DOI: 10.1002/14651858.CD002098.pub2
2. Huang Z, Tao H, Meng Q, et al. Effects of telecare intervention on glycemic control in type 2 diabetes: a systematic review and meta-analysis of randomized controlled trials. European Journal of Endocrinology 2015;172(3):R93-R101.
3. Liang X, Wang Q, Yang X, et al. Effect of mobile phone intervention for diabetes on glycaemic control: A meta-analysis. Diabetic Medicine 2011;28(4):455-63.
4. Marcolino MS, Maia JX, Alkmim MBM, et al. Telemedicine application in the care of diabetes patients: Systematic review and meta-analysis. PLoS ONE 2013;8(11).
5. Pal K, Eastwood SV, Michie S, et al. Computer-based interventions to improve self-management in adults with type 2 diabetes: A systematic review and meta-analysis. Diabetes Care 2014;37(6):1759-66.
6. Polisena J, Tran K, Cimon K, et al. Home telehealth for diabetes management: a systematic review and meta-analysis. Diabetes Obes Metab 2009;11(10):913-30.
7. Qi L, Liu Q, Qi X, et al. Effectiveness of peer support for improving glycaemic control in patients with type 2 diabetes: a meta-analysis of randomized controlled trials. BMC Public Health 2015;15(1):1-11.
8. Saffari M, Ghanizadeh G, Koenig HG. Health education via mobile text messaging for glycemic control in adults with type 2 diabetes: A systematic review and meta-analysis. Primary Care Diabetes 2014.
9. Su D, Zhou J, Kelley MS, et al. Does telemedicine improve treatment outcomes for diabetes? A meta-analysis of results from 55 randomized controlled trials. Diabetes Research and Clinical Practice;116:136-48.
10. Tao D, Or CK. Effects of self-management health information technology on glycaemic control for patients with diabetes: a meta-analysis of randomized controlled trials. Journal of Telemedicine and Telecare 2013;19(3):133-43.
11. Verhoeven F, Tanja-Dijkstra K, Nijland N, et al. Asynchronous and synchronous teleconsultation for diabetes care: a systematic literature review. J Diabetes Sci Technol 2010;4(3):666-84.
12. Zhai Y-k, Zhu W-j, Cai Y-l, et al. Clinical- and Cost-effectiveness of Telemedicine in Type 2 Diabetes Mellitus: A Systematic Review and Meta-analysis. Medicine 2014;93(28):e312.
13. Zhang X, Yang S, Sun K, et al. How to achieve better effect of peer support among adults with type 2 diabetes: A meta-analysis of randomized clinical trials. Patient Education and Counseling;99(2):186-97.

Appendix 11a: Summary of effect sizes comparing meta-analysis from other studies and from network meta-analysis on glycemic control from the current study

Comparison	Traditional meta-analysis			Network meta-analysis (current study)	
	Study, year (Reference)	Effect size (95% CI)	Trials included, n	Effect size (95% CI)	Trials included, n
Tele-education vs usual care	Saffari, 2014	-0.60 (-0.83 to -0.36)	10	-0.37 (-0.59 to -0.15)	26
Telemonitoring vs usual care	Polisena, 2009	-0.21 (-0.35 to -0.08)	21	-0.40 (-0.70 to -0.11)	14
	Liang, 2011	-0.81 (-1.11 to -0.50)	10		
	Flodgren, 2015	0.03 (-0.28 to 0.34)	2		
Telecase-management vs usual care	Huang, 2015	-0.54 (-0.75 to -0.34)	18	-0.24 (-0.61 to 0.14)	8
	Pal, 2014	-0.21 (-0.37 to -0.05)	12		
Teleconsultation vs usual care	Verhoeven, 2007	0.03 (-0.31 to 0.24)	6	-0.71 (-1.23 to -0.20)	7
	Flodgren, 2015	-0.27 (-0.34 to -0.19)	3		
Telementoring vs usual care	Qi, 2015	-0.57 (-0.78 to -0.36)	13	-0.28 (-0.61 to 0.04)	11
	Zhang, 2016	-0.16 (-0.25 to -0.07)	20		
Telecase-management & Telemonitoring vs usual care	Flodgren, 2015	-0.57 (-0.73 to -0.41)	11	-0.58 (-0.96 to -0.19)	9

Appendix 11b: Summary of effect sizes comparing meta-analysis from other studies and pairwise meta-analysis on glycemic control from the current study

Comparison	Study, year (Reference)	Effect size (95% CI)	Trials included, n	Effect size (95% CI)	Trials included, n
Telemedicine studies vs usual care	Marcolino, 2013	-0.44 (-0.61 to -0.26)	6	-0.42 (-0.63 to -0.21)	92
	Tao, 2013	-0.36 (-0.48 to -0.24)	32		
	Zhai, 2014	-0.37 (-0.49 to -0.25)	35		
	Flodgren, 2015	-0.31 (-0.37 to -0.24)	16		
	Su, 2016	-0.63	31		

Figure S1: Flow chart depicts the process of study selection

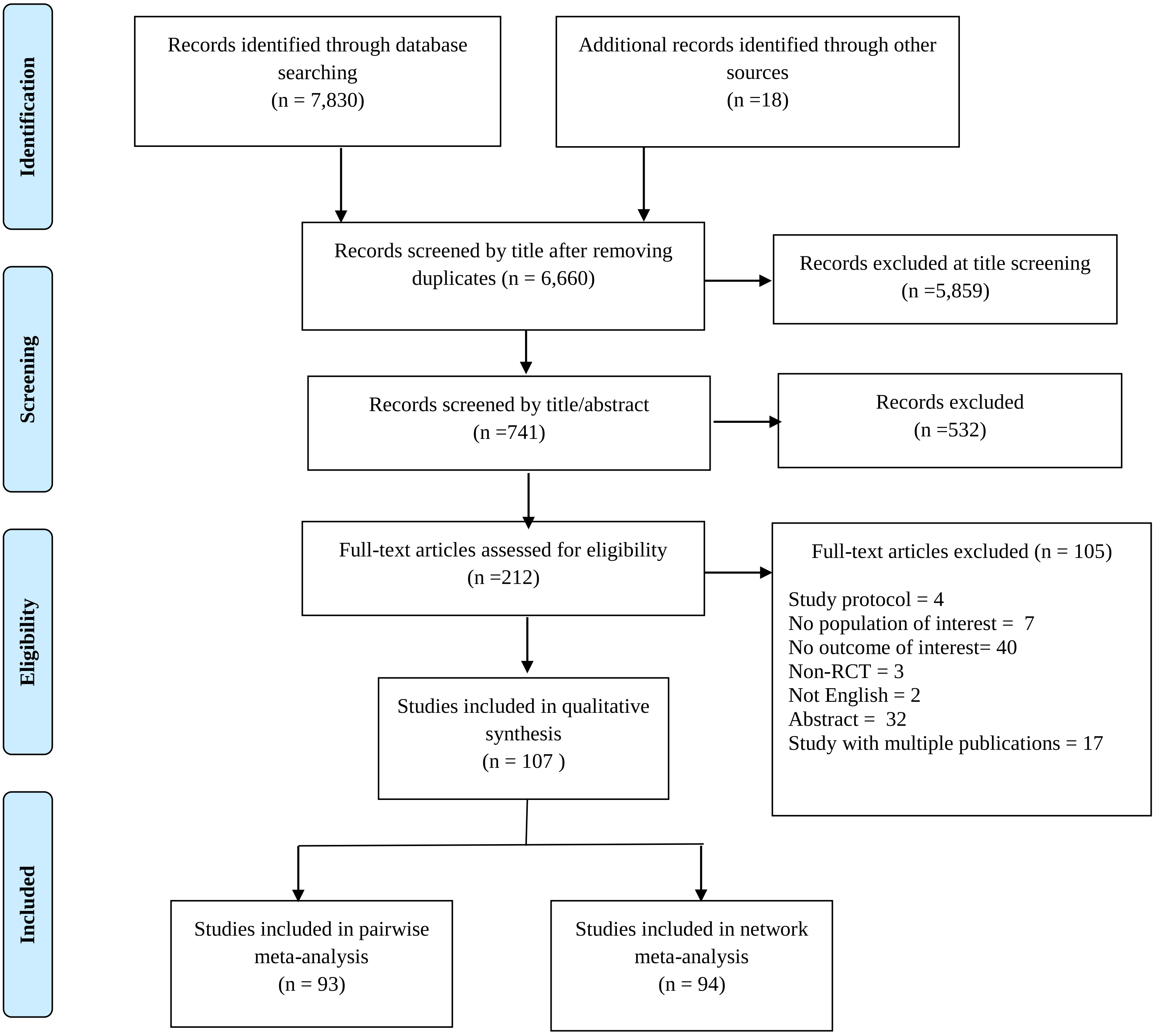

Figure S2: Risk of bias assessment appraisal of randomized controlled studies on telemedicine using the Cochrane risk of bias tool

To assess the risk of bias for studies included in the current study, we used the Cochrane Risk of Bias tool. This tool assessed the specific bias of seven domains including methods for generating the random sequence, allocation concealment, blinding of participants and investigators, blinding of outcome assessment, incompleteness of outcome data and selective outcome reporting. The adjudication of the risk of bias is achieved by answering pre-specified questions about the methods reported by each study in relation to the risk domain, and results are represented in a risk of bias table.

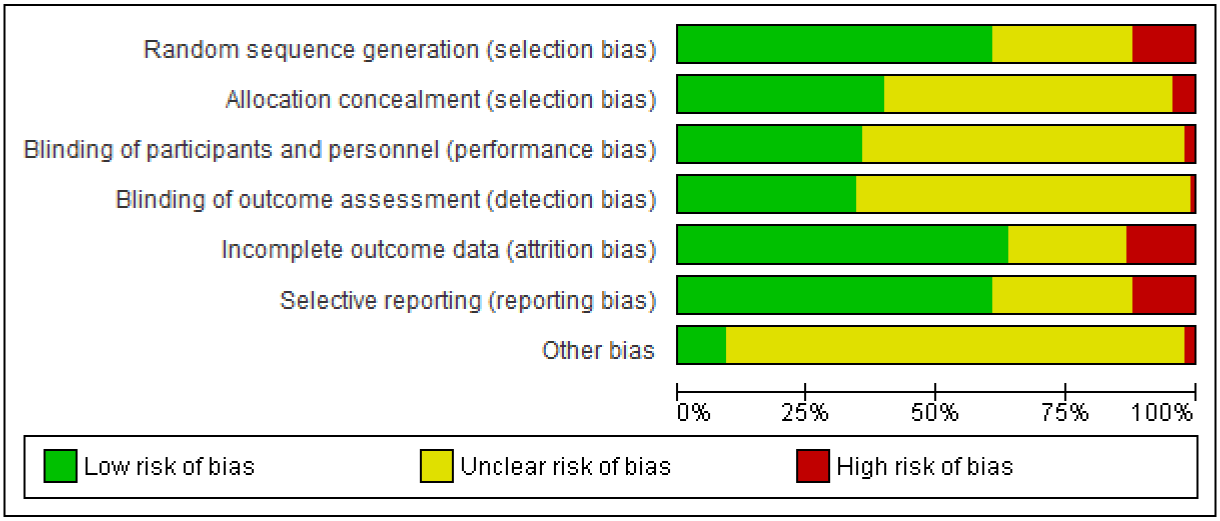

Figure S3 Evidence network for outcomes assessed

The width of lines for each connection in the evidence network is proportional to the number of randomized controlled studies that compared each pair of treatment. The sizes of nodes are proportional to the number of patients. The numbers represent the number of randomized controlled studies which contribute to the direct comparison.

Figure S3a: Evidence network of treatment comparison for primary outcome

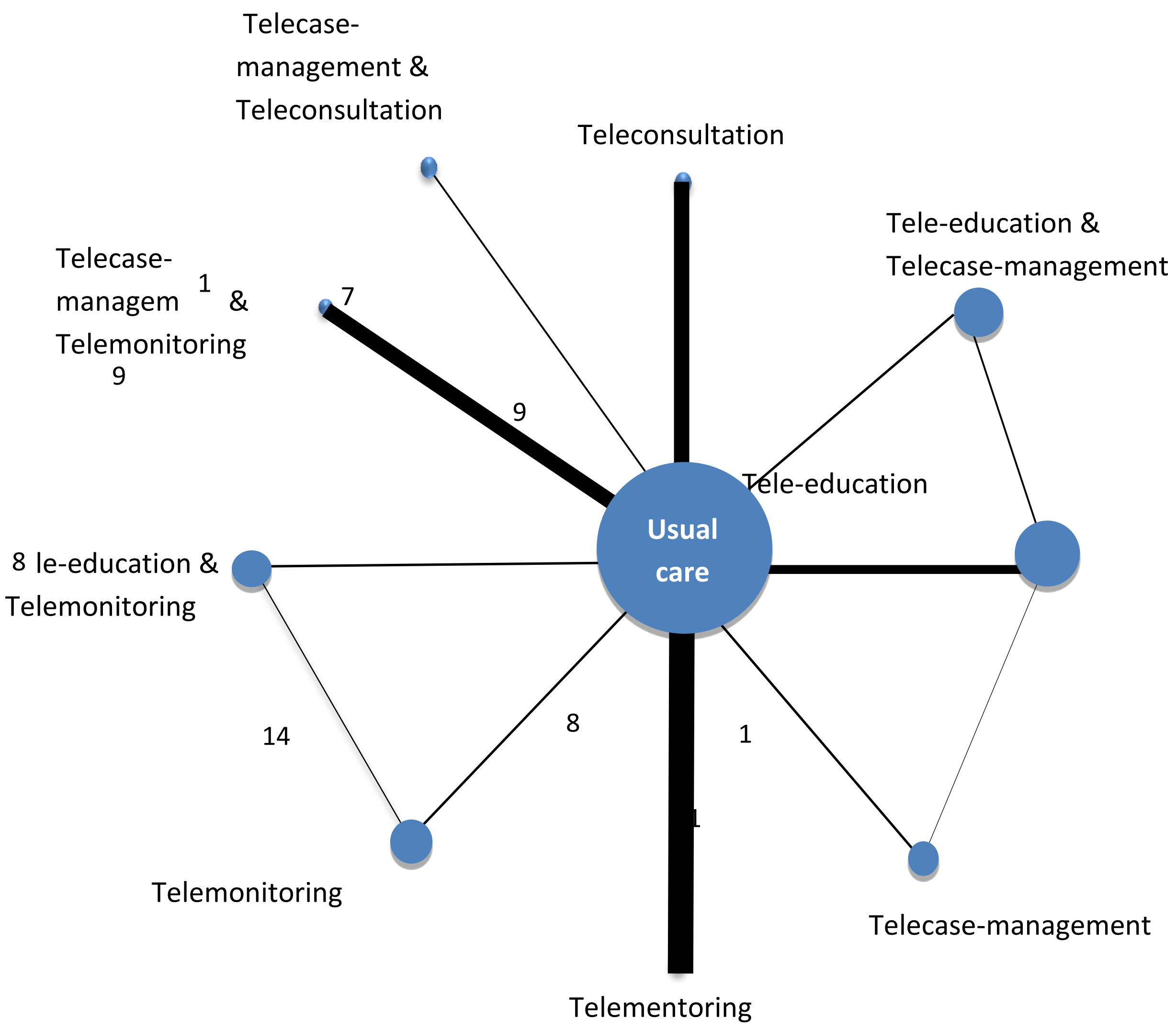

The tau value for network meta-analysis for is 0.52 (moderate heterogeneity)

Figure S3b: Network of treatment comparison for secondary outcomes

Fasting plasma glucose

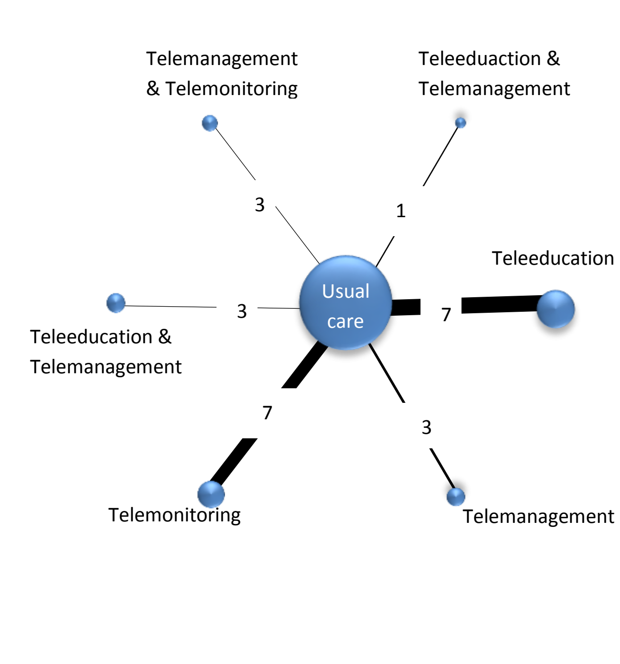

Systolic blood pressure

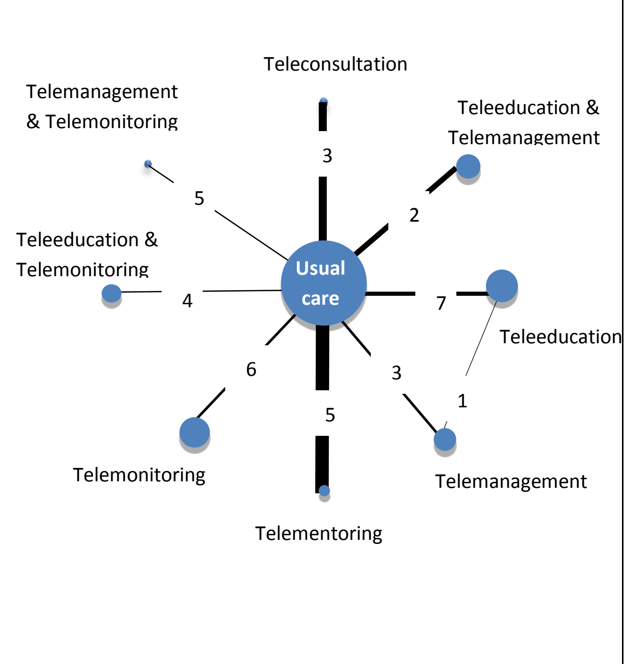

Diastolic blood pressure

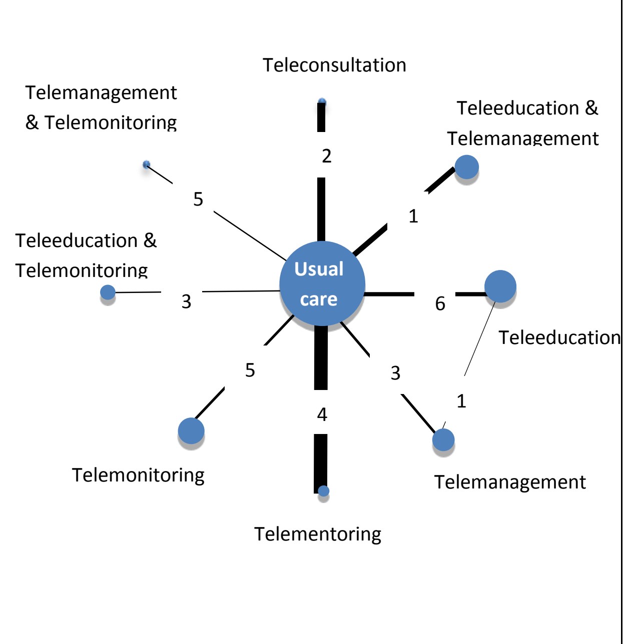

Body Mass Index

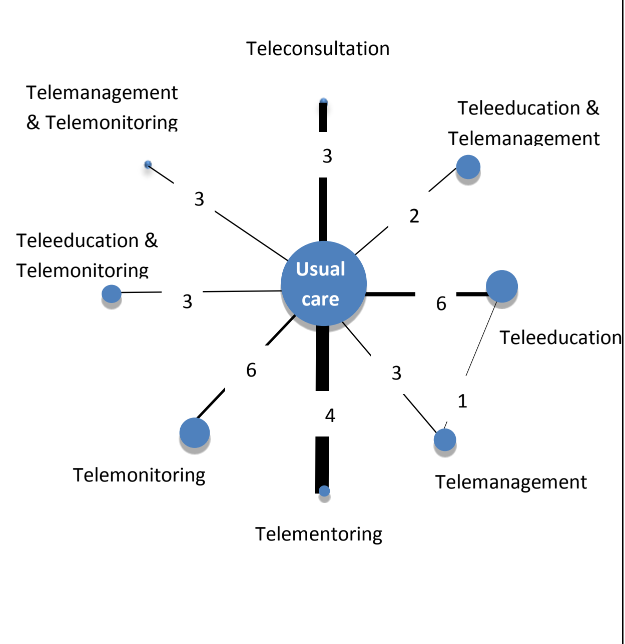

The tau value for network meta-analysis for each outcomes (an estimate of a single heterogeneity variance for the

Total cholesterol

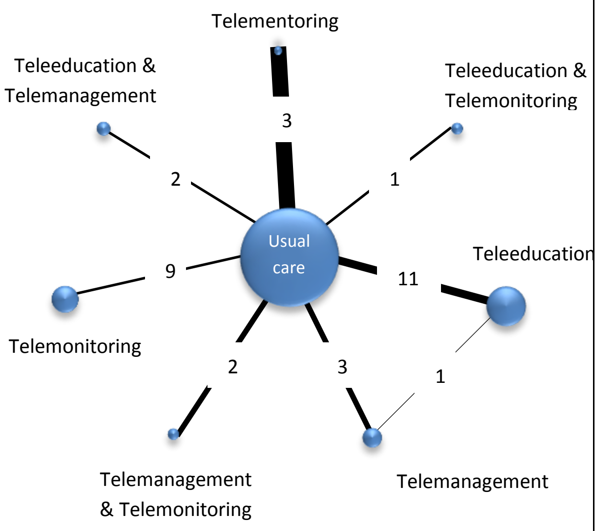

Low density lipoprotein

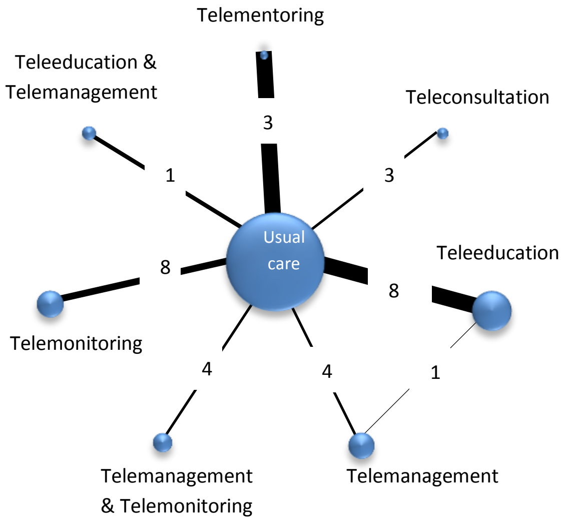

High density lipoprotein

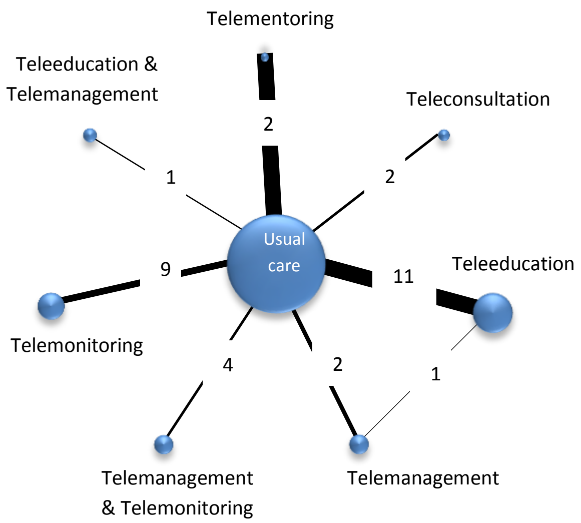

Triglycerides

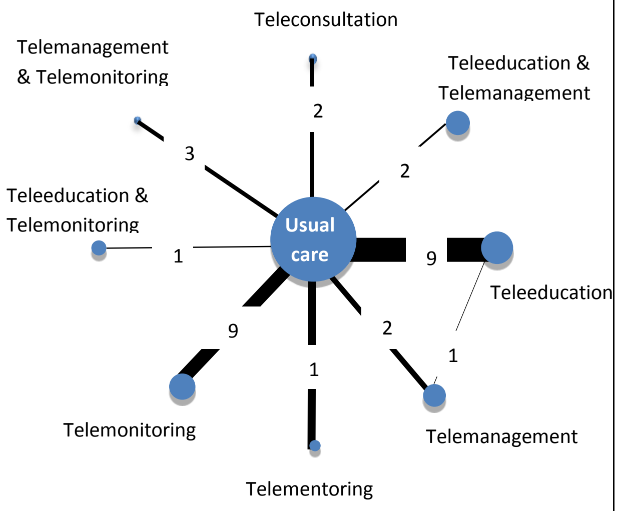

whole network) are as follow: fasting plasma glucose: 0.66 (moderate heterogeneity) systolic blood pressure τ = 1.80 (high heterogeneity) ; diastolic blood pressure, τ=1.22 (high heterogeneity); BMI, τ=0.85 (high heterogeneity); total cholesterol, τ=0.44 (moderate heterogeneity); low-density lipoprotein, τ= 0.15 (moderate heterogeneity); high-density lipoprotein , τ= 0.06 (low heterogeneity); triglycerides , τ= 0.06 (low heterogeneity).

Figure S4: Surface under cumulative ranking curve of the various treatments for glycosylated hemoglobin. Ranking indicates probability to be the best treatment, second best and so on among the different telemedicine interventions evaluated

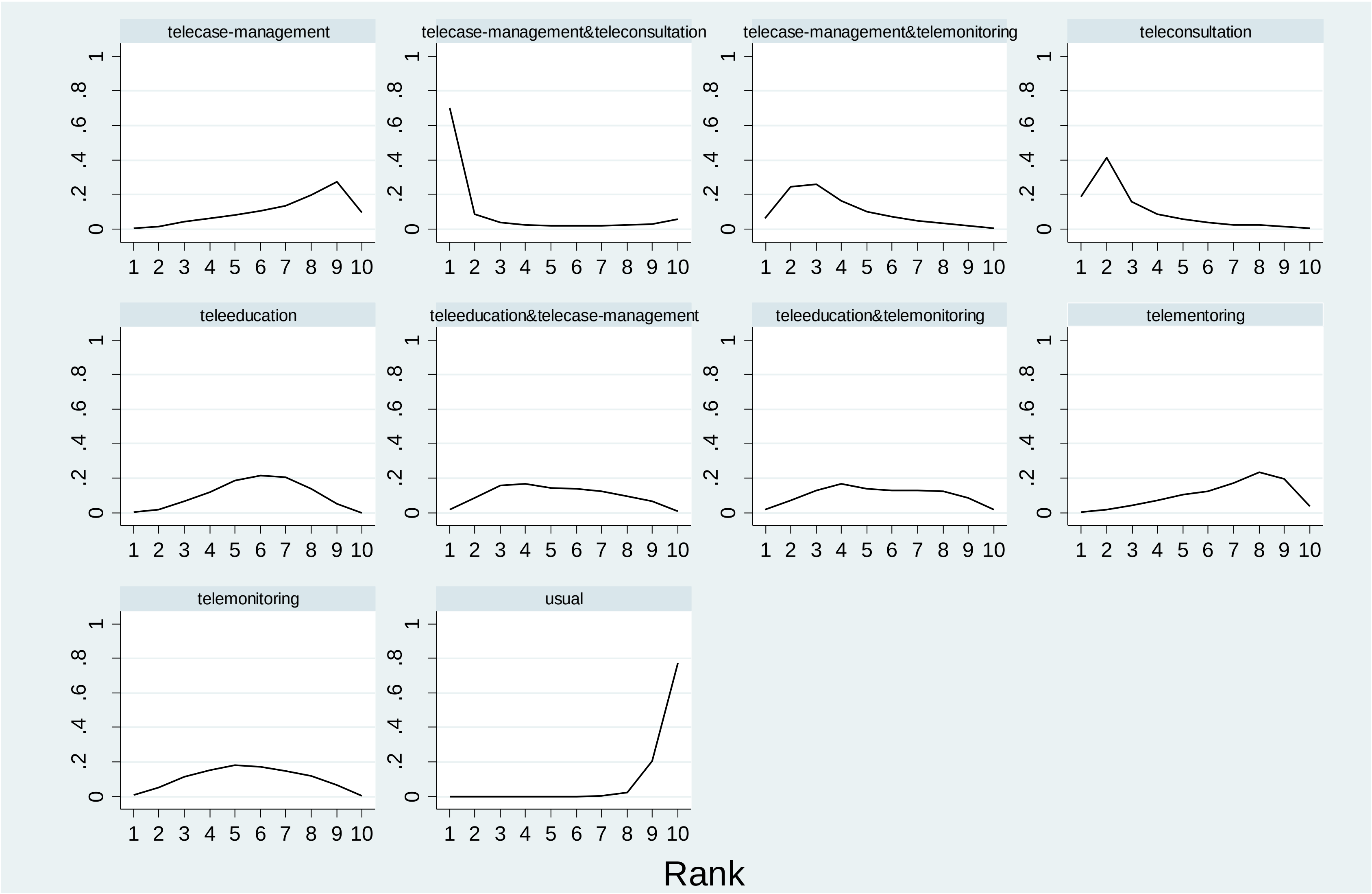

Figure S5: Comparison adjusted funnel plot

Each observation represents the difference between the study estimate and its direct meta-analysis mean effect. The red line represents the null hypothesis, that is study specific effect sizes do not differ from the respective comparison specific pooled effect estimates. Studies on the right hand side ‘over-estimate’ the effect of active treatment. All comparisons are active treatment against usual care.

Figure S5a: Comparison adjusted funnel plot for glycosylated hemoglobin.

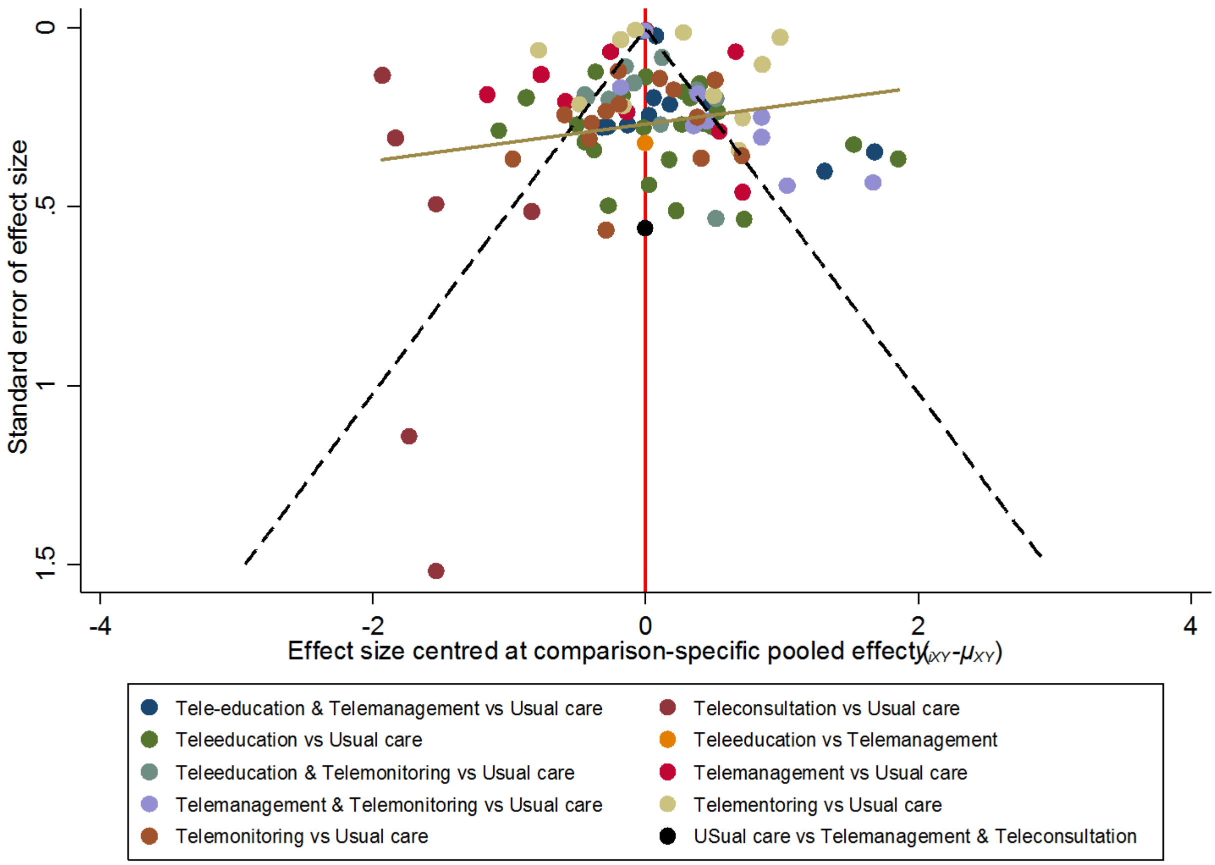

Figure S5b: Comparison adjusted funnel plot for secondary outcomes

Fasting plasma glucose

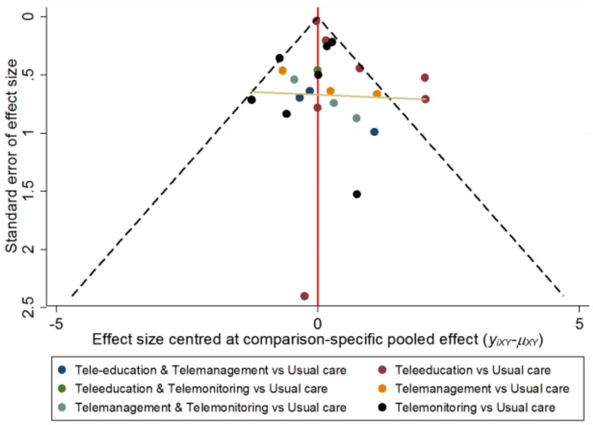

Systolic blood pressure

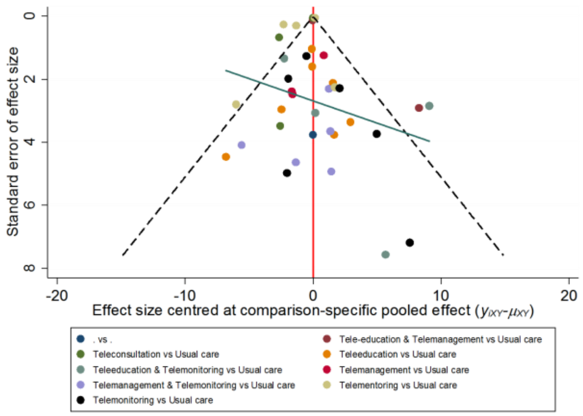

Diastolic blood pressure

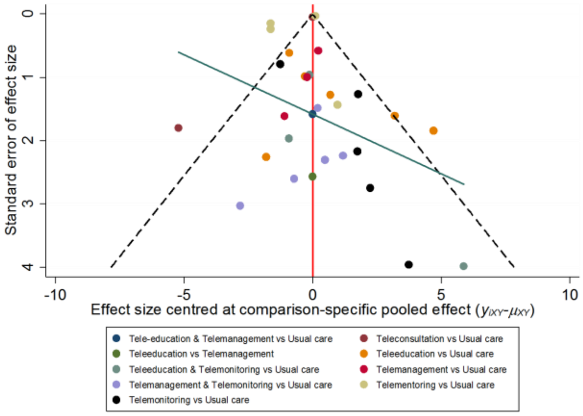

Body Mass Index

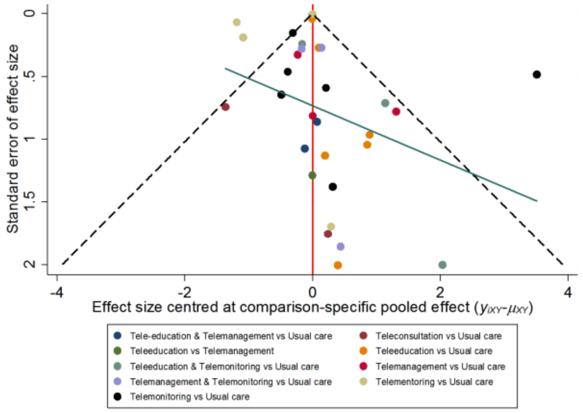

Total cholesterol	Low density lipoprotein
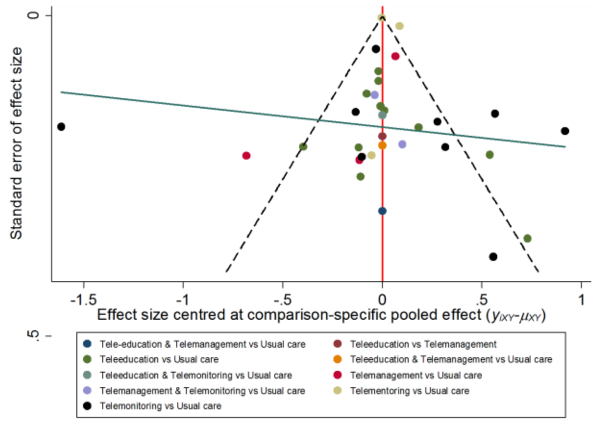	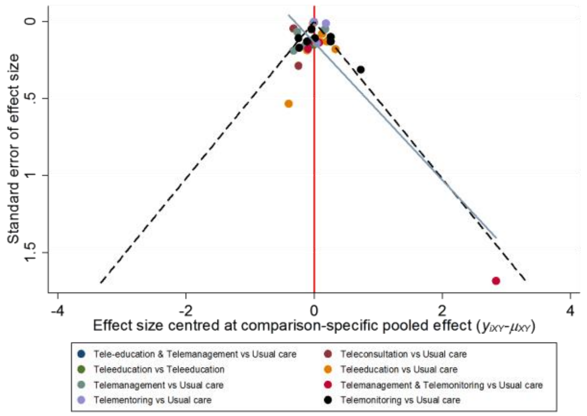
**High density lipoprotein**	**Triglycerides**
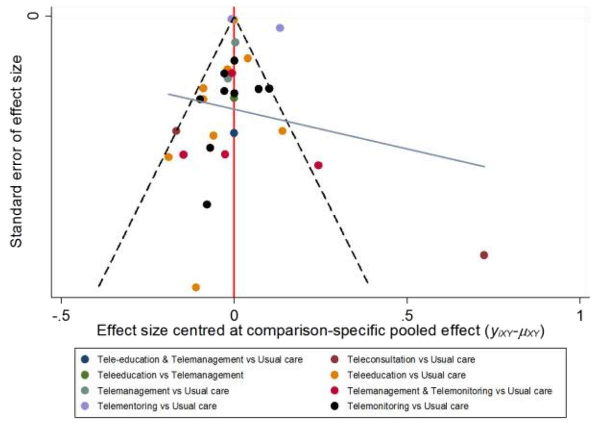	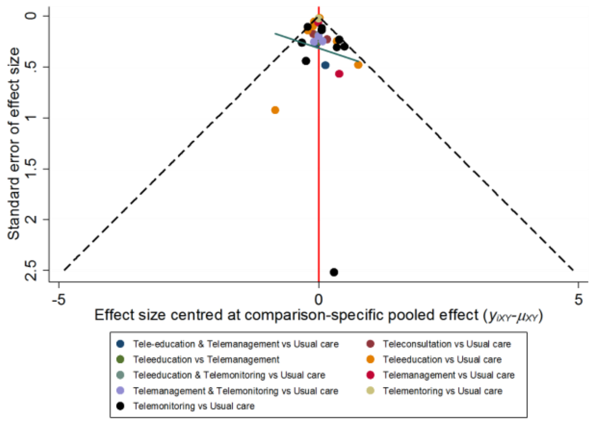

eReferences: List of studies included in the current review

1. Anderson DR, Christison-Lagay J, Villagra V, et al. Managing the space between visits: A randomized trial of disease management for diabetes in a community health center. J Gen Intern Med 2010;25(10):1116-22.

2. Anderson-Loftin W, Barnett S, Bunn P, et al. Soul Food Light: Culturally Competent Diabetes Education. Diabetes Educator 2005;31(4):555-63.

3. Anzaldo-Campos MC, Contreras S, Vargas-Ojeda A, et al. Dulce Wireless Tijuana: A Randomized Control Trial Evaluating the Impact of Project Dulce and Short-Term Mobile Technology on Glycemic Control in a Family Medicine Clinic in Northern Mexico. Diabetes Technology & Therapeutics 2016;18(4):240-51.

4. Arora S, Peters AL, Burner E, et al. Trial to examine text message-based mHealth in emergency department patients with diabetes (TExT-MED): a randomized controlled trial. Ann Emerg Med 2014;63(6):745-54 e6.

5. Aubert RE, Herman WH, Waters J, et al. Nurse case management to improve glycemic control in diabetic patients in a health maintenance organization: A randomized, controlled trial. Ann Intern Med 1998;129(8):605-12.

6. Avdal EU, Kizilci S, Demirel N. The effects of web-based diabetes education on diabetes care results: A randomized control study. CIN - Computers Informatics Nursing 2011;29(2):101-06.

7. Bell AM, Fonda SJ, Walker MS, et al. Mobile phone-based video messages for diabetes self-care support. Journal of Diabetes Science and Technology 2012;6(2):310-19.

8. Blackberry ID, Furler JS, Best JD, et al. Effectiveness of general practice based, practice nurse led telephone coaching on glycaemic control of type 2 diabetes: The Patient Engagement and Coaching for Health (PEACH) pragmatic cluster randomised controlled trial. BMJ (Online) 2013;347(7926).

9. Boaz M, Hellman K, Wainstein J. An automated telemedicine system improves patient-reported well-being. Diabetes Technology & Therapeutics 2009;11(3):181-86.

10. Bond GE, Burr R, Wolf FM, et al. The Effects of a Web-Based Intervention on the Physical Outcomes Associated with Diabetes Among Adults Age 60 and Older: A Randomized Trial. Diabetes Technology & Therapeutics 2007;9(1):52-59.

11. Browning C, Chapman A, Yang H, et al. Management of type 2 diabetes in China: the Happy Life Club, a pragmatic cluster randomised controlled trial using health coaches. BMJ Open 2016;6(3):e009319.

12. Bujnowska-Fedak MM, Puchala E, Steciwko A. The impact of telehome care on health status and quality of life among patients with diabetes in a primary care setting in Poland. Telemedicine journal and e-health 2011;17(3):153-63.

13. Capozza K, Woolsey S, Georgsson M, et al. Going Mobile With Diabetes Support: A Randomized Study of a Text Message–Based Personalized Behavioral Intervention for Type 2 Diabetes Self-Care. Diabetes Spectrum 2015;28(2):83-91.

14. Carter EL, Nunlee-Bland G, Callender C. A Patient-Centric, Provider-Assisted Diabetes Telehealth Self-management Intervention for Urban Minorities. Perspectives in Health Information Management 2011;8(1):1-9.

15. Castelnuovo G, Manzoni GM, Cuzziol P, et al. TECNOB Study: Ad Interim Results of a Randomized Controlled Trial of a Multidisciplinary Telecare Intervention for Obese Patients with Type-2 Diabetes. Clinical Practice and Epidemiology in Mental Health : CP & EMH 2011;**7**:44-50.

16. Chan JC, Sui Y, Oldenburg B, et al. Effects of telephone-based peer support in patients with type 2 diabetes mellitus receiving integrated care: a randomized clinical trial. JAMA Intern Med 2014;174(6):972-81.

17. Chen SY, Chang YH, Hsu HC, et al. One-year efficacy and safety of the telehealth system in poorly controlled type 2 diabetic patients receiving insulin therapy. Telemedicine journal and e-health 2011;17(9):683-87.

18. Cho J, Chang S, Kwon H, et al. Long-term effect of the Internet-based glucose monitoring system on HbA1c reduction and glucose stability: a 30-month follow-up study for diabetes management with ubiquitous medical care system. Diabetes Care 2006;29(12):2625-31.

19. Cho J, Lee H, Lim D, et al. Mobile communication using a mobile phone with a glucometer for glucose control in Type 2 patients with diabetes: as effective as an Internet-based glucose monitoring system. Journal of Telemedicine & Telecare 2009;15(2):77-82.

20. Cho JH, Kwon HS, Kim HS, et al. Effects on diabetes management of a health-care provider mediated, remote coaching system via a PDA-type glucometer and the Internet. Journal of Telemedicine and Telecare 2011;17(7):365-70.

21. Ciemins E, Coon P, Peck R, et al. Using telehealth to provide diabetes care to patients in rural Montana: Findings from the Promoting Realistic Individual Self-Management Program (PRISM). Diabetes Technology and Therapeutics 2013;15(SUPPL.1):S70.

22. Crowley MJ, Powers BJ, Olsen MK, et al. The Cholesterol, Hypertension, And Glucose Education (CHANGE) study: Results from a randomized controlled trial in African Americans with diabetes. American Heart Journal;166(1):179-86.e2.

23. Dale J, Caramlau I, Sturt J, et al. Telephone peer-delivered intervention for diabetes motivation and support: The telecare exploratory RCT. Patient Education & Counseling 2009;75(1):91-98.

24. Dang TNT, Deoisres W, Keeratiyutawong P, et al. Effectiveness of a diabetes self-management support intervention in Vietnamese adults rith type 2 diabetes. Journal of Science, Technology and Humanities 2013;11(1):13-23.

25. Davis RM, Hitch AD, Salaam MM, et al. TeleHealth improves diabetes self-management in an underserved community: diabetes TeleCare. Diabetes Care 2010;33(8):1712-7.

26. Del Prato S, Nicolucci A, Lovagnini-Scher AC, et al. Telecare provides comparable efficacy to conventional self-monitored blood glucose in patients with type 2 diabetes titrating one injection of insulin glulisine-the ELEONOR study. Diabetes Technology and Therapeutics 2012;14(2):175-82.

27. Earle KA, Istepanian RS, Zitouni K, et al. Mobile telemonitoring for achieving tighter targets of blood pressure control in patients with complicated diabetes: a pilot study. Diabetes Technol Ther 2010;12(7):575-9.

28. Faridi Z, Liberti L, Shuval K, et al. Evaluating the impact of mobile telephone technology on type 2 diabetic patients' self-management: The NICHE pilot study. Journal of Evaluation in Clinical Practice 2008;14(3):465-69.

29. Glasgow R, La Chance P, Toobert D, et al. Long-term effects and costs of brief behavioural dietary intervention for patients with diabetes delivered from the medical office. Patient Education & Counseling 1997;32(3):175-84.

30. Glasgow RE, Nutting PA, Toobert DJ, et al. Effects of a brief computer-assisted diabetes self-management intervention on dietary, biological and quality-of-life outcomes. Chronic Illness 2006;2(1):27-38.

31. Glasgow R, Kurz D, King D, et al. Outcomes of minimal and moderate support versions of an Internet-based diabetes self-management support program. Journal of General Internal Medicine 2010;25(12):1315-22.

32. Goodarzi M, Ebrahimzadeh I, Rabi A, et al. Impact of distance education via mobile phone text messaging on knowledge, attitude, practice and self efficacy of patients with type 2 diabetes mellitus in Iran. Journal of Diabetes and Metabolic Disorders 2012;11(1):1-8.

33. Graziano JA, Gross CR. A randomized controlled trial of an automated telephone intervention to improve glycemic control in type 2 diabetes. Advances in Nursing Science 2009;32(3):E42-E57.

34. Greenwood DA, Blozis SA, Young HM, et al. Overcoming Clinical Inertia: A Randomized Clinical Trial of a Telehealth Remote Monitoring Intervention Using Paired Glucose Testing in Adults With Type 2 Diabetes. Journal of medical Internet research 2015;17(7):e178.

35. Heisler M, Vijan S, Makki F, et al. Diabetes control with reciprocal peer support versus nurse care management: a randomized trial. Ann Intern Med 2010;153(8):507-15.

36. Holmen H, Torbjørnsen A, Wahl KA, et al. A mobile health intervention for self-management and lifestyle change for persons with type 2 diabetes, Part 2: One-year results from the Norwegian Randomized Controlled Trial RENEWING HEALTH. JMIR mHealth uHealth 2014;2(4):e57.

37. Hsu WC, Lau KHK, Huang R, et al. Utilization of a cloud-based diabetes management program for insulin initiation and titration enables collaborative decision making between healthcare providers and patients. Diabetes technology & therapeutics 2016;18(2):59-67.

38. Huizinga MM, Gebretsadik T, Garcia Ulen C, et al. Preventing glycaemic relapse in recently controlled type 2diabetes patients: A randomised controlled trial. Diabetologia 2010;53(5):832-39.

39. Hussein WI, Hasan K, Jaradat AA. Effectiveness of mobile phone short message service on diabetes mellitus management; the SMS-DM study. Diabetes Research and Clinical Practice 2011;94(1):e24-e26.

40. Islam SMS, Niessen LW, Ferrari U, et al. Effects of Mobile Phone SMS to Improve Glycemic Control Among Patients With Type 2 Diabetes in Bangladesh: A Prospective, Parallel-Group, Randomized Controlled Trial. Diabetes Care 2015;38(8):e112-e13.

41. Karhula T, Vuorinen A-L, Rääpysjärvi K, et al. Telemonitoring and Mobile Phone-Based Health Coaching Among Finnish Diabetic and Heart Disease Patients: Randomized Controlled Trial. Journal of Medical Internet Research 2015;17(6):e153.

42. Keyserling TC, Samuel-Hodge CD, Ammerman AS, et al. A Randomized Trial of an Intervention to Improve Self-Care Behaviors of African-American Women With Type 2 Diabetes. Impact on physical activity 2002;25(9):1576-83.

43. Kim H, Oh J. Adherence to diabetes control recommendations: impact of nurse telephone calls. Journal of Advanced Nursing 2003;44(3):256-61.

44. Kim SI, Kim HS. Effectiveness of mobile and internet intervention in patients with obese type 2 diabetes. International Journal of Medical Informatics 2008;77(6):399-404.

45. Kim MT, Han HR, Song HJ, et al. A community-based, culturally tailored behavioral intervention for Korean Americans with type 2 diabetes. Diabetes Educator 2009;35(6):986-94.

46. Kim H-S, Sun C, Yang SJ, et al. Randomized, Open-label, Parallel Group Study to Evaluate the Effect of Internet-based Glucose Management System on Subjects with Diabetes in China. Telemedicine and e-Health 2016.

47. Kwon HS, Cho JH, Kim HS, et al. Development of web-based diabetic patient management system using short message service (SMS). Diabetes Res Clin Pract 2004;66 Suppl 1:S133-7.

48. Lee T-I, Yeh Y-T, Liu C-T, et al. Development and evaluation of a patient-oriented education system for diabetes management. International Journal of Medical Informatics 2007;76(9):655-63.

49. Lee JY, Lee SWH, Nasir NH, et al. Diabetes telemonitoring reduces the risk of hypoglycaemia during Ramadan: a pilot randomized controlled study. Diabetic Medicine 2015;32(12):1658-61.

50. Leichter SB, Bowman K, Adkins RA, et al. Impact of remote management of diabetes via computer: the 360 study--a proof-of-concept randomized trial. Diabetes Technol Ther 2013;15(5):434-8.

51. Leu MG, Norris TE, Hummel J, et al. A randomized, controlled trial of an automated wireless messaging system for diabetes. Diabetes Technol Ther 2005;7(5):710-18.

52. Lim S, Kang SM, Shin H, et al. Improved glycemic control without hypoglycemia in elderly diabetic patients using the ubiquitous healthcare service, a new medical information system. Diabetes Care 2011;34(2):308-13.

53. Lim S, Kang SM, Kim KM, et al. Multifactorial intervention in diabetes care using real-time monitoring and tailored feedback in type 2 diabetes. Acta Diabetologica 2016;53(2):189-98.

54. Liou JK, Soon MS, Chen CH, et al. Shared care combined with telecare improves glycemic control of diabetespatients in a rural, underserved community. Journal of Diabetes Science and Technology 2014;20(2):175-78.

55. Lorig K, Ritter PL, Villa F, et al. Spanish diabetes self-management with and without automated telephone reinforcement: two randomized trials. Diabetes Care 2008;31(3):408-14.

56. Long JA, Jahnle EC, Richardson DM, et al. Peer Mentoring and Financial Incentives to Improve Glucose Control in African American Veterans: A Randomized, Controlled Trial. Annals of internal medicine 2012;156(6):416-24.

57. Luley C, Blaik A, Reschke K, et al. Weight loss in obese patients with type 2 diabetes: Effects of telemonitoring plus a diet combination - The Active Body Control (ABC) Program. Diabetes Research & Clinical Practice 2011;91(3):286-92.

58. Maljanian R, Grey N, Staff I, et al. Intensive telephone follow-up to a hospital-based disease management model for patients with diabetes mellitus. Disease Management 2005;8(1):15-25.

59. McKay H, Glasgow RE, Feil EG, et al. Intenet-based diabetes self-management and support: Initial outcome from the Diabetes Network Project. Rehab ilitation Psychology 2002;47(1):31-48.

60. McMahon GT, Gomes HE, Hickson Hohne S, et al. Web-based care management in patients with poorly controlled diabetes. Diabetes Care 2005;28(7):1624-9.

61. McMahon GT, Fonda SJ, Gomes HE, et al. A randomized comparison of online- and telephone-based care management with internet training alone in adult patients with poorly controlled type 2 diabetes. Diabetes technology & therapeutics 2012;14(11):1060-67.

62. Nagrebetsky A, Larsen M, Craven A, et al. Stepwise self-titration of oral glucose-lowering medication using a mobile telephone-based telehealth platform in type 2 diabetes: A feasibility trial in primary care. Journal of Diabetes Science and Technology 2013;7(1):123-34.

63. Nesari M, Zakerimoghadam M, Rajab A, et al. Effect of telephone follow-up on adherence to a diabetes therapeutic regimen. Japan journal of nursing science : JJNS 2010;7(2):121-28.

64. Nicolucci A, Cercone S, Chiriatti A, et al. A Randomized Trial on Home Telemonitoring for the Management of Metabolic and Cardiovascular Risk in Patients with Type 2 Diabetes. Diabetes technology & therapeutics 2015;17(8):563-70.

65. Noh JH, Cho YJ, Nam HW, et al. Web-based comprehensive information system for self-management of diabetes mellitus. Diabetes technology & therapeutics 2010;12(5):333-37.

66. Oh JA, Kim HS, Yoon KH, et al. A telephone-delivered intervention to improve glycemic control in type 2 diabetic patients. Yonsei Medical Journal 2003;44(1):1-8.

67. Or C, Tao D. A 3-Month Randomized Controlled Pilot Trial of a Patient-Centered, Computer-Based Self-Monitoring System for the Care of Type 2 Diabetes Mellitus and Hypertension. Journal of Medical Systems 2016;40(4):1-13.

68. Orsama AL, Lahteenmaki J, Harno K, et al. Active assistance technology reduces glycosylated hemoglobin and weight in individuals with type 2 diabetes: Results of a theory-based randomized trial. Diabetes Technology and Therapeutics 2013;15(8):662-69.

69. Peimani M, Rambod C, Omidvar M, et al. Effectiveness of short message service-based intervention (SMS) on self-care in type 2 diabetes: A feasibility study. Primary Care Diabetes.

70. Piette JD, Weinberger M, McPhee SJ, et al. Do automated calls with nurse follow-up improve self-care and glycemic control among vulnerable patients with diabetes? The American journal of medicine 2000;108(1):20-7.

71. Piette JD, Weinberger M, Kraemer FB, et al. Impact of automated calls with nurse follow-up on diabetes treatment outcomes in a Department of Veterans Affairs health care system: A randomized controlled trial. Diabetes Care 2001;24(2):202-08.

72. Piette JD, Richardson C, Himle J, et al. A randomized trial of telephonic counseling plus walking for depressed diabetes patients. Medical Care 2011;49(7):641-48.

73. Plotnikoff R, Karunamuni N, Courneya K, et al. The Alberta Diabetes and Physical Activity Trial (ADAPT): A randomized trial evaluating theory-based interventions to increase physical activity in adults with type 2 diabetes. Annals of Behavioral Medicine 2013;45(1):45-56.

74. Pressman AR, Kinoshita L, Kirk S, et al. A novel telemonitoring device for improving diabetes control: protocol and results from a randomized clinical trial. Telemed J E Health 2014;20(2):109-14.

75. Quinn CC, Shardell MD, Terrin ML, et al. Cluster-randomized trial of a mobile phone personalized behavioral intervention for blood glucose control. Diabetes Care 2011;34(9):1934-42.

76. Ralston JD, Hirsch IB, Hoath J, et al. Web-Based Collaborative Care for Type 2 Diabetes: A pilot randomized trial. Diabetes Care 2009;32(2):234-39.

77. Rasmussen O, Lauszus F, Loekke M. Telemedicine compared with standard care in type 2 diabetes mellitus: A randomized trial in an outpatient clinic. Journal of Telemedicine and Telecare 2015.

78. Rodríguez-Idígoras MI, Sepúlveda-Muñoz J, Sánchez-Garrido-Escudero R, et al. Telemedicine influence on the follow-up of type 2 diabetes patients. Diabetes technology & therapeutics 2009;11(7):431-37.

79. Sacco WP, Malone JI, Morrison AD, et al. Effect of a brief, regular telephone intervention by paraprofessionals for type 2 diabetes. Journal of Behavioral Medicine 2009;32(4):349-59.

80. Samuel-Hodge CD, Keyserling TC, France R, et al. A Church-based Diabetes Self-management Education Program for African Americans With Type 2 Diabetes. Preventing Chronic Disease 2006;3(3):A93.

81. Schillinger D, Handley M, Wang F, et al. Effects of Self-Management Support on Structure, Process, and Outcomes Among Vulnerable Patients With Diabetes: A three-arm practical clinical trial. Diabetes Care 2009;32(4):559-66.

82. Shahid M, Mahar S, Shaikh S, et al. Mobile phone intervention to improve diabetes care in rural areas of Pakistan: a randomized controlled trial. J Coll Physicians Surg Pak 2015;25(3):166-71.

83. Shea S, Weinstock RS, Teresi JA, et al. A randomized trial comparing telemedicine case management with usual care in older, ethnically diverse, medically underserved patients with diabetes mellitus: 5 year results of the IDEATel study. J Am Med Inform Assoc 2009;16(4):446-56.

84. Shetty A, Chamukuttan S, Nanditha A, et al. Reinforcement of adherence to prescription recommendations in Asian Indian diabetes patients using short message service (SMS)-a pilot study. J Assoc Physicians India 2011;59:711-14.

85. Smith SA, Shah ND, Bryant SC, et al. Chronic care model and shared care in diabetes: randomized trial of an electronic decision support system. Mayo Clinic proceedings 2008;83(7):747-57.

86. Spencer MS, Rosland A-M, Kieffer EC, et al. Effectiveness of a Community Health Worker Intervention Among African American and Latino Adults With Type 2 Diabetes: A Randomized Controlled Trial. American journal of public health 2011;101(12):2253-60

87. Steventon A, Bardsley M, Doll H, et al. Effect of telehealth on glycaemic control: analysis of patients with type 2 diabetes in the Whole Systems Demonstrator cluster randomised trial. BMC Health Services Research 2014;14:334-34.

88. Stone RA, Rao RH, Sevick MA, et al. Active care management supported by home telemonitoring in veterans with type 2 diabetes: The DiaTel randomized controlled trial. Diabetes Care 2010;33(3):478-84.

89. Stone RA, Sevick MA, Rao RH, et al. The Diabetes Telemonitoring Study Extension: an exploratory randomized comparison of alternative interventions to maintain glycemic control after withdrawal of diabetes home telemonitoring. J Am Med Inform Assoc 2012;19(6):973-9.

90. Tang PC, Overhage JM, Chan AS, et al. Online disease management of diabetes: Engaging and motivating patients online with enhanced resources-diabetes (EMPOWER-D), a randomized controlled trial. Journal of the American Medical Informatics Association 2013;20(3):526-34.

91. Tildesley HD, Mazanderani AB, Chan JHM, et al. Efficacy of A1C reduction using internet intervention in patients with type 2 diabetes treated with insulin. Canadian Journal of Diabetes 2011;35(3):250-53.

92. Thom DH, Ghorob A, Hessler D, et al. Impact of Peer Health Coaching on Glycemic Control in Low-Income Patients With Diabetes: A Randomized Controlled Trial. Annals of Family Medicine 2013;11(2):137-44.

93. Tjam EY, Sherifali D, Steinacher N, et al. Physiological outcomes of an Internet disease management program vs. In-person counselling: A randomized, controlled trial. Canadian Journal of Diabetes 2006;30(4):397-405.

94. Wakefield BJ, Holman JE, Ray A, et al. Effectiveness of home telehealth in comorbid diabetes and hypertension: a randomized, controlled trial. Telemedicine journal and e-health 2011;17(4):254-61.

95. Waki K, Fujita H, Uchimura Y, et al. DialBetics: A Novel Smartphone-based Self-management Support System for Type 2 Diabetes Patients. Journal of Diabetes Science and Technology 2014;8(2):209-15.

96. Walker EA, Shmukler C, Ullman R, et al. Results of a successful telephonic intervention to improve diabetes control in urban adults: A randomized trial. Diabetes Care 2011;34(1):2-7.

97. Wayne N, Perez DF, Kaplan DM, et al. Health Coaching Reduces HbA1c in Type 2 Diabetic Patients From a Lower-Socioeconomic Status Community: A Randomized Controlled Trial. Journal of medical Internet research 2015;17(10):e224.

98. Whitlock W, Brown A, Moore K, et al. Telemedicine improved diabetic management. Military medicine 2000;165(8):579.

99. Whittemore R, Melkus GD, Sullivan A, et al. A nurse-coaching intervention for women with type 2 diabetes. Diabetes Educator 2004;30(5):795-804.

100. Williams ED, Bird D, Forbes AW, et al. Randomised controlled trial of an automated, interactive telephone intervention (TLC Diabetes) to improve type 2 diabetes management: baseline findings and six-month outcomes. BMC public health 2012;12

101. Wolever RQ, Dreusicke M, Fikkan J, et al. Integrative health coaching for patients with type 2 diabetes: A randomized clinical trial. Diabetes Educator 2010;36(4):629-39.

102. Wong FK, Mok MP, Chan T, et al. Nurse follow-up of patients with diabetes: randomized controlled trial. J Adv Nurs 2005;50(4):391-402.

103. Yoo HJ, Park MS, Kim TN, et al. A Ubiquitous Chronic Disease Care system using cellular phones and the internet. Diabetic Medicine 2009;26(6):628-35.

104. Yoon KH, Kim HS. A short message service by cellular phone in type 2 diabetic patients for 12 months. Diabetes Research and Clinical Practice 2008;79(2):256-61.

105. Young RJ, Burns E, Taylor J, et al. Pro-active call center treatment support (PACCTS) to improve glucose control in type 2 diabetes - A randomized controlled trial. Diabetes Care 2005;28(2):278-82.

106. Zhou P, Xu L, Liu X, et al. Web-based telemedicine for management of type 2 diabetes through glucose uploads: a randomized controlled trial. International Journal of Clinical and Experimental Pathology 2014;7(12):8848-54.

107. Zolfaghari M, Mousavifar SA, Haghani H. Mobile phone text messaging and Telephone follow-up in type 2 diabetic patients for 3 months: A comparative study. Journal of Diabetes and Metabolic Disorders 2012;11(1).
